# Proteomic analysis of rat colonic mucosa following acupuncture treatment for irritable bowel syndrome with diarrhea

**DOI:** 10.1371/journal.pone.0273853

**Published:** 2022-09-12

**Authors:** Jing Liu, Rui Peng, Qian Tan, Bocun Li, Jianyi Chen, Guangya Liu, Yawen Wang, Chao Li, Jia Li, Hua Wang

**Affiliations:** Acupuncture-Moxibustion and Orthopedic College, Hubei University of Chinese Medicine, Wuhan, Hubei, China; University of Illinois at Chicago, UNITED STATES

## Abstract

To investigate the molecular pathological mechanisms of irritable bowel syndrome with diarrhea (IBS-D) and elucidate the effects of acupuncture on IBS-D colonic mucosa protein abundance in rats, a label-free high-throughput liquid chromatography-tandem mass spectrometry (LC-MS)-based proteomics analysis was used to survey the global changes of colonic mucosa proteins between different groups. Sixteen Sprague-Dawley (SD) male rats were randomly divided into four groups: the control group (C); the IBS-D model group (M); the syndrome differentiation acupuncture group (SD) and the traditional acupuncture group (T). IBS-D model rats were obtained using the CAS (chronic acute combining stress model) method. Comparative bioinformatics analysis of the proteomic data was analyzed using MaxQuant software, Perseus software, online tools DAVID, VENNY and STRING. Functional enrichment and network analyses revealed a close relationship between IBS-D and several biological processes including energy metabolism, muscular excitation/contraction, and both traditional acupuncture and syndrome differentiation acupuncture can reverse the impairments of normal energy metabolism. Moreover, the syndrome differentiation acupuncture can regulate the protein cluster relating inflammation, wound repair and cell protection against oxidative stress which is associated with acupuncture analgesic effect. Differentially expressed proteins Atp5a1 and Bpnt1 were selected as representative proteins and subjected to western blotting. In conclusion, our study provides further insight into the pathological and molecular mechanisms of IBS-D and acupuncture treatments, and serves as an experimental basis for clinical applications.

## Introduction

Irritable bowel syndrome is a common digestive disorder in clinical. According to the main symptoms of stomachache, diarrhea, abdominal distension and altered bowel habit, IBS can be divided into four subtypes: IBS with constipation (IBS-C), IBS with diarrhea (IBS-D), mixed IBS (IBS-M), and unsubtyped IBS (IBS-U). IBS-D is the most common subtype among them [1]. The pathogenesis of IBS is not well understood, and it is considered to be the result of multiple factors such as gastrointestinal motility abnormality, visceral paresthesia, abnormal brain-intestinal regulation, inflammation and mental psychology [2, 3]. Psychological abnormality is also closely associated with IBS-D, which can alter the brain-intestinal interactions and may exacerbate IBS. The acute or chronic stress enhances the intestinal permeability weakening of the tight junctions and increasing bacterial translocation into the intestinal wall [4, 5]. Meta-analysis revealed that the anxiety level of IBS-C and IBS-D patients is higher than healthy people, and IBS-D patients showed the highest level of depression mood [6]. Besides, IBS patients tend to have elevated trauma, life stress, relationship conflicts, and emotional avoidance, which few therapies directly target [7]. Modern medicine mainly use psychobehavioral therapy combined with drug therapy to achieve the purpose of treating IBS. However, it is difficult to meet the patients’ needs and drug therapy always accompany with adverse drug reactions including dry eyes and mouth, blurred vision, dizziness and vomiting [8–10]. Under these circumstances, it is of great value to seek the help of alternative and complementary medicine in treating IBS. Thakur’s study demonstrated that brief emotional awareness and expression training that targeted trauma and emotional conflicts could reduce somatic symptoms and improve quality of life in patients with IBS [7]. This emotion-focused approach may be considered an additional treatment option for IBS reduced somatic symptoms and improved quality of life in patients with IBS.

Acupuncture is a traditional therapy applied for thousands of years, and it was also a powerful non-drug therapy which is used extensively in Oriental Medicine and has emerged as an important modality of complementary and alternative therapy to western medicine [11]. Electroacupuncture (EA) has been widely used clinically to treat gastrointestinal and mental illnesses [12, 13]. Clinical trials have proven that acupuncture is effective in improving IBS symptoms in four aspects of the regulatory effects including gastrointestinal motility, the gastrointestinal barrier, visceral sensitivity, and the brain-gut axis [12]. According to TCM, one characteristic of acupuncture is that it has opposite regulatory effects during different physiological conditions. For example, acupuncture may promote gastric peristalsis in subjects with low initial gastric motility, but it may suppress peristalsis in subjects with active initial motility [12, 13]. Iwa et al. suggested that in rats, acupuncture at Zusanli (ST-36) not only promoted gastric peristalsis, but also inhibited the acceleration of stress-induced colonic transit due to restraint [14]. The gastrointestinal barrier is a component of GI defense, protects the epithelium from harmful microbes and toxins [15]. Researches demonstrated that acupuncture may be helpful in restoring GI barrier injury by regulating the neuron-endocrineimmune system and antagonizing the inflammatory response. In rats with stress-induced gastric ulcers, EA at Zusanli (ST-36) aided in the repair of the stomach mucosa by increasing the concentration of epidermal growth tissue. Besides, EA at bilateral points of Zusanli (ST-36) and shangjuxu (ST-37) significantly attenuated chronic visceral hypersensitivity that was induced by neonatal colon irritation in rats [16, 17]. Moreover, acupuncture has been acknowledged as an effective treatment for abdominal pain in IBS patients [18]. The brain-gut axis is a bridge that connects the CNS and GI tract [19]. Hui KK etc. conducted a functional magnetic resonance imaging (fMRI) study of the human brain. The result demonstrated that manual acupuncture at ST-36 modulated neural activity at multiple levels in the cerebro-cerebellar and limbic systems [20].

Viscera syndrome differentiation and treating disease from the root are the essence of TCM. IBS-D was series sign and symptoms arising from stress reaction or irregular movement of small intestine and large intestine. Traditional Chinese medicine considers the etiology of IBS as lack of spirit preservation, liver depression, weakness of the spleen and the stomach and deficiency of Yang. Therefore, in the acupuncture treatment of IBS-D, we should treat both exterior and interior and regulate organs functions. Zusanli (ST-36), Neiguan (PC-6), Shangjuxu (ST-37), Zhongwan (CV-12), and Tianshu (ST-25) are the primary acupoints used in the treatment of GI disorders [14]. In our latest work, we use the syndrome differential acupuncture (manual acupuncture at Zusanli (ST-36), Neiguan (PC-6) and Guanyuan (CV-4)) to treat IBS-D. We have analyzed the principle of choosing acupoints in the treatment of IBS-D using the data mining method. The analysis was based on the randomised or quasi-randomised controlled trials of acupuncture treatment on IBS-D in databases CNKI, WanFang Data, VIP, PubMed, Cochrane Library and Embase. Results showed that Shangjuxu (ST-37)-Tianshu (ST-25)-Zusanli (ST-36) is used most widely in IBS-D treatment [21]. Therefore, we choose acupoints Shangjuxu (ST-37), Tianshu (ST-25), Zusanli (ST-36) as the control of syndrome differential acupoints. The previous research demonstrated that syndrome differential acupuncture can effectively relieve anxiety or depression, regulate gastrointestinal motility and alleviate visceral hypersensitivity, and the therapeutic effect is better than the traditional acupoint selection method of treating IBS-D (unpublished).

Although acupuncture has been used as an appropriate treatment for IBS, the underlying mechanisms of acupuncture on gastrointestinal function have not been clearly understood. Besides, the pathophysiology of IBS-D is also not well understood due to numerous factors playing multiple roles in disease development. Under these circumstances, proteomics is regarded as a powerful tool to explore the underlying mechanisms of IBS-D and acupuncture effects. Based on the previous researches, we conducted the label-free quantitative proteomic analyses to identify key proteins potentially associated with IBS-D and acupuncture effects, to compare the differences between syndrome differential acupuncture and traditional acupuncture, and to provide an experimental basis for clinical applications of acupuncture in treating IBS-D.

## Materials and methods

### Animal treatment

A total of 16 Sprague-Dawley male rats in 3-month-old were obtained from China Three Gorges University [Experimental animal production license number: SCXK2017-0012]. All the rats were raised in the individually ventilated cages with the temperature between 20°C to 25°C and the humidity between 45% and 55%. Light was provided from 8 am to 8 pm to simulate the circadian rhythms while food and water was offered sufficiently. All animal treatments were approved by the Animal Ethics Committee of Hubei Province, No.00287332.

After 1 week of adaptation, the rats were randomly divided into four groups: the control group (group C), the IBS-D model group (group M), the syndrome differentiation acupuncture group (group SD) and the traditional acupuncture group (group T). Rats in group C were raised normally without any treatments. The IBS-D model rats were obtained using CAS (chronic acute combining stress model) method [22]. The CAS group was exposed to the following seven different stressors: water deprivation for 24 hours; food deprivation for 24 hours; painful tail pinch for 1 minute; 5 minutes exposure to a 45°C environment; swimming in 4°C water for 3 minutes; day and night inversion for 12 hours/12 hours; horizontal vibration (120 rpm) for 45 minutes. All stress protocols were applied at random every 7 days for 3 weeks, and no specific stressor was repeated on 2 consecutive days.

The visceral sensitivity was measured by the abdominal withdrawal reflex (AWR) test to evaluate the IBS-D model. Rats in group M were raised without any treatments after modeling while rats in group SD and group T started acupuncture treatment at the same time of modeling once a day for 28 days. The process of the experiment was shown in S1 Fig. The body temperature, tongue temperature and the weight of rats were measured every three days during the model making and acupuncture procedures to make sure that the physiological indicators were normal. All efforts were made to minimize suffering.

### Visceral sensitivity test

The visceral sensitivity of rats in different groups was measured using the abdominal withdrawl reflex (AWR) describing as following before and after model-building [23]. Firstly, connect the sphygmomanometer, the needle tubing and the home-made finger-cot airbag using a t-branch pipe. Then put the rat in the fixator and keep it awake. Insert the airbag into the anus of the rat at a depth of approximately 5 centimeters. After adapting to the environment, pumping to expand the intestinal tract gradually while observe the number of the sphygmomanometer to evaluate the AWR. Record the pressure thresholds (mm Hg) which lead to the raising of abdomen and arching of back. The thresholds were measured for three times and take an average.

### Acupuncture treatment

Sterile acupuncture needles [size: 0.30mm×13mm (diameter: 0.30 millimeter, length: 13 millimeter); made by Suzhou Acupuncture & Moxibustion Appliance Co., Lid, Suzhou, P.R. China] were used during the acupuncture treatment. Acupoints were determined according to *Experimental acupuncture* and the acupuncture point map of rats made by Yin CS etc. [24]. For the SD group, acupuncture needles were inserted at the bilateral Zusanli (ST-36), Neiguan (PC-6) and Guanyuan (CV-4) at to a depth of 2 mm. Then twirl and rotate the needle backward and forward continuously with the frequency 2 times per second for 1 minute. The same manipulation was done after every 5 minutes for three times until withdrawing the needle. Zusanli (ST-36) is located on the lower lateral of knee-joint, 5 millimeters below the caput fibulae; Neiguan (PC-6) is located on the inner side of the rat foreleg, 3 millimeters from the wrist joints between the radioulnar suture; Guanyuan (CV-4) is located about 25 millimeters below the rat navel, 15 millimeters above the pubic symphysis, on the 3/5 point of the connecting line between navel and superior border of pubic symphysis. For the T group, the same procedure was applied to acupoints Shangjuxu (ST-37), Tianshu (ST-25) and Zusanli (ST-36). Shangjuxu (ST-37) is located in the hindlimb of rats, approximately 5 millimeters below Zusanli (ST-36). Tianshu (ST-25) is located in the abdomen, 2 inches away from the anterior median line at the same level of umbilical. The acupuncture treatment was performed every day except Sunday for 28 days.

### Sample preparation and protein extraction

At the end of the acupuncture treatment, rats from four groups were fasted overnight. Then all the rats were sacrificed by cervical dislocation, and 2-cm segments of the proximal colon from descending colon were cut, washed with PBS containing the protease inhibitor and opened longitudinally [25]. Mucosa were obtained using a sharp blade to avoid contamination from the muscle layer, frozen in the liquid nitrogen and stored at -80°C for protein extraction.

About 20 mg tissues in each sample were used for protein extraction. Frozen tissues were washed twice with cold PBS and grinded using a pestle in liquid nitrogen. Add 200μL of the lysis buffer (1 mL RIPA; 10μL PMSF) to the prepared tissues and homogenized for 2 minutes, then kept at 4°C for 10 minutes. Next, the mixture was centrifuged at 12000 rpm at 4°C for 20 minutes and the supernatant containing the tissue protein was collected. 100μL of the supernatant was then mixed with 400μL ice-cold methanol and incubated at -20°C overnight. Subsequently, centrifuged at 12000 rpm for 20 minutes at 4°C and collected the precipitation. After that, the precipitation were treated with 120μL urea, 28μL H_2_0, 8μL 1 M NH_4_HCO_3_, 4μL 1 M dithiothreitol for 2 hours at 37°C followed by 8μL 1 M iodacetamide at room temperature in dark place for 1 hour. The proteins were then digested with trypsin (Sigma, Cat. No. T7409, enzyme/protein ratio of 1:25 w/w) at 37°C overnight. At last, all the protein samples were desalted using the C18 column before the LC-MS/MS analysis.

### LC-MS/MS analysis

After desalting, the peptides were separated by a Nano flow Ultimate 3000 HPLC (Dionex Corp, Sunnyvale, CA) coupled online to a Q-Exactive Plus mass spectrometer (Thermo Fisher Scientific, Waltham, MA, USA). The eluted peptides were loaded onto a C18-reversed phase column with mobile phase buffers A (5% acetonitrile, 0.1% formic acid) and B (95% acetonitrile, 0.1% formic acid) at a flow rate of 0.2 mL/min. Subsequently, the peptides were eluted with a 75 min linear gradient: 0 min, 5% B; 3 min, 5% B; 48 min, 50% B; 55 min, 80% B; 60 min, 80% B; 65 min, 5% B; 75min, 5% B. Eluted peptides were analyzed by DDA method. A wash column method was executed between samples to avoid protein retaining in the column. Data acquisition was achieved in a positive polarity mode with a survey scan 200–2000 m/z and the survey scans were acquired at a resolution of 70,000 while for MS/MS spectra this was set to 17,500. Automatic gain control was used to ensure that the Orbitrap mass analyzer always filled with the optimum number of ions for generation of MS/MS spectra. The intensity threshold, which defines the minimum precursor intensity to trigger an MS2 scan, was set to 1.6×10^5^. In order to increase the likely coverage of lower abundant precursor ions, a 10 s dynamic exclusion list was employed. The mass spectrometry proteomics data have been deposited to the ProteomeXchange Consortium via the PRIDE [26] partner repository with the dataset identifier PXD025558.

### Protein identification and quantitation

The MS/MS data were analyzed using MaxQuant software (version1.6.1.0) [27]. The peak lists were searched against the UniProt Rattus norvegicus protein database. For the searches, precursor mass tolerance of 20 ppm, fragment mass tolerance of 0.1 Da. Trypsin/P with up to two missed cleavage were allowed. Variable modifications were set to: oxidation (M) and acetylation (protein N-term). Fixed modifications were set for carbamidomethylation of cysteine. A protein level false-discovery rate (FDR) of 1% was set to filter the result. Label-free quantitation (LQF) of protein was also performed in MaxQuant. The minimum number of peptides that has to be available in pair-wise comparisions between two samples for a protein was 1. LFQ min number of neighbors was set 3 and LFQ average number of neighbors was 6.

### Bioinformatics analysis

The Perseus software (version 1.6.7.0) was used to identify proteins differentially expressed between different samples. Comparative bioinformatics analysis of differentially expressed proteins (DEPs) was then performed in a stepwise manner as following. The Reference Sequence (RefSeq) IDs obtained after the proteomics search by maxquant software were converted into gene symbol using online tool DAVID Bioinformatics Resources 6.8 [Nature Protocols 2009; 4(1):44 & Nucleic Acids Res. 2009;37(1):1.]. Venn diagram was obtained using VENNY to compare the number and overlapping relationships of DE proteins between different groups (Available from: http://bioinfogp.cnb.csic.es/tools/venny/index.html). Subsequently, Gene Ontology (GO) enrichment analysis and pathway analysis of differentially expressed genes was implemented by DAVID [Nature Protocols 2009; 4(1):44 & Nucleic Acids Res. 2009;37(1):1.]. GO terms and enriched pathways with corrected Pvalue less than 0.05 were considered significantly enriched by differential expressed proteins. The pathway analysis results were visualized using heatmaps generated by Heml heat map illustrator program [28]. Protein-protein interaction networks were analyzed by the STRING (Search Tool for the Retrieval of Interacting Genes/Proteins) system 11.0 (http://stringdb.org/) and Cytoscape software (version 3.7.1).

### Western blot

Protein expression levels were verified using western blot analysis. The protein sample from the previous step was separated on 10% SDS-PAGE gel and then electrotransferred to the polyvinylidene fluoride membrane (Merck Millipore, Billerica, MA). After being sealed with 5% bovine serum albumin at room temperature for 2 hours, the membrane was incubated overnight with anti-ATP5a1 (1:2000, proteintech) and anti-BPNT1(1:2000, proteintech) at 4°C, and the membrane was hybridized with secondary antibodies bound to horseradish peroxidase. After incubating the membrane with enhanced chemiluminescence reagent, the protein band images were collected and analyzed by molecular image and chemical XRS image system (Bio-Rad laboratory). The density value was scanned and counted by Image Lab software, and β-actin (1:2000, Abcam, UK) was used as the internal standard, that is, the gray value of the target protein / the total gray value of the internal standard.

### Statistical analysis

Standard selection criteria to identify differentially expressed genes are established at |Fold change| ≥ 1.2 and P < 0.05. Student’s t-test (two-tailed) was used for data analysis this study. A P value of less than 0.05 was considered statistically significant.

## Results

### The visceral sensitivity of rats in different groups

**Fig 1:**
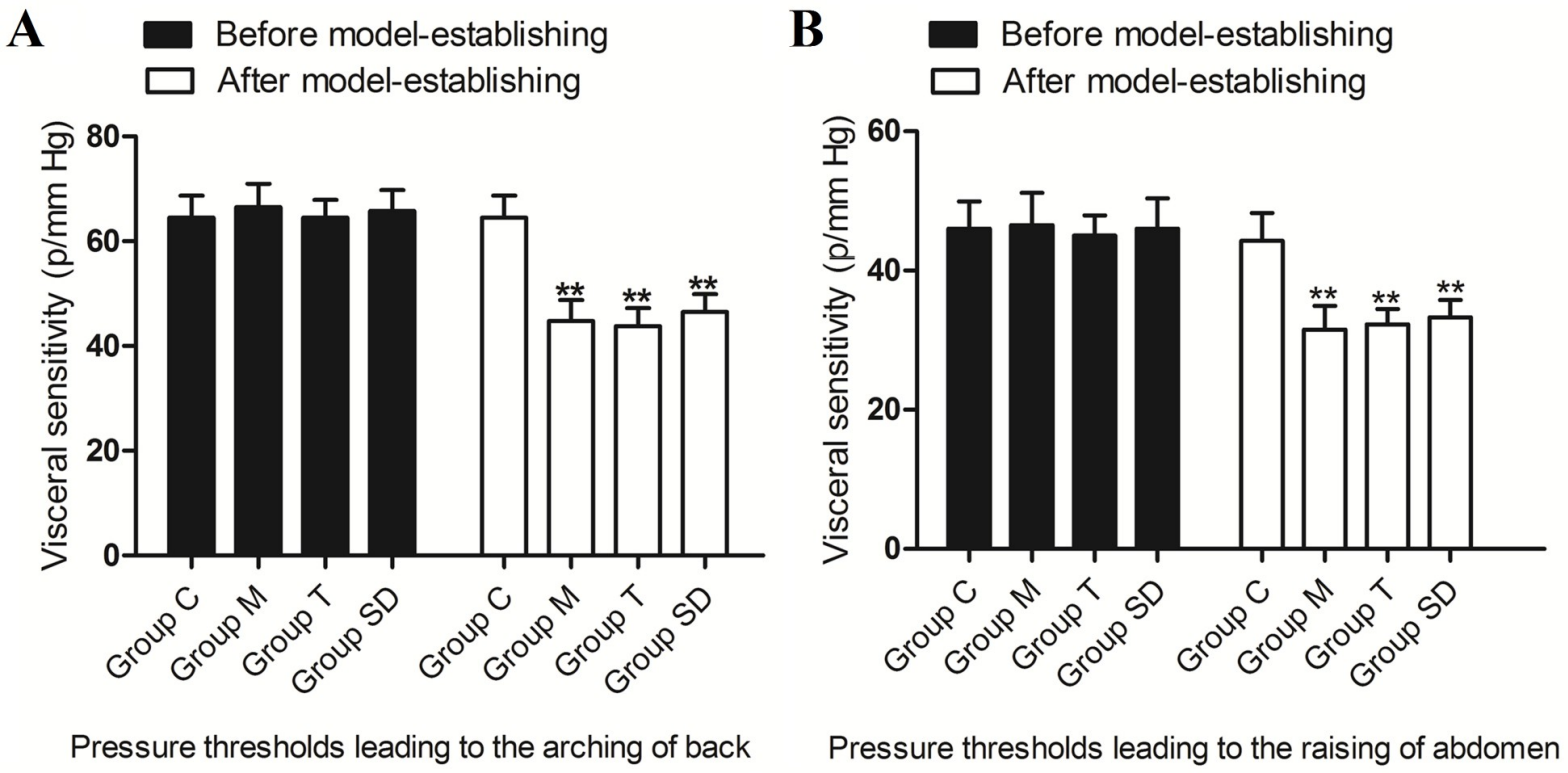
Comparison of the visceral sensitivity of rats in different groups. A: Pressure thresholds leading to the arching of back; B: Pressure thresholds leading to the raising of abdomen.**P<0.01.

The result showed no significant differences in the pressure thresholds leading to the raising of abdomen and arching of back among different groups before establishing of the IBS-D model (P>0.05). After the model is established, the pressure thresholds leading to the raising of abdomen and arching of back in IBS-D rats were decreased significantly when compared to the control group (P<0.01), which indicated the successful of the IBS-D model (Fig 1).

### Identification of differentially expressed proteins between different groups

To explore the biological mechanism of IBS-D and acupuncture effects, the differentially expressed (DE) proteins between different samples were analyzed. LC-MS/MS was employed to obtain the proteome profiles of each group. In total, 1106 unique proteins were identified across all samples. Label-free quantification and differential abundance analysis was performed using MaxQuant and Perseus softwares. We identified 47 proteins changing significantly between the control and model samples. Of these, 14 were up-regulated and 33 were down-regulated in group M compared with group C. Direct comparisons revealed significantly higher levels of 27 proteins, and lower levels of 16 proteins in group T compared to group M. While in group SD, there were 56 DE proteins, of these 37 were down-regulated and 19 were up-regulated compared to group M; see Table 1.

**Table 1:** The number of DE proteins between different groups.

Comparision	Up-regulated	Down-regulated
M vs C	14	33
T vs M	16	27
SD vs M	37	19
T vs SD	11	6

**Fig 2:**
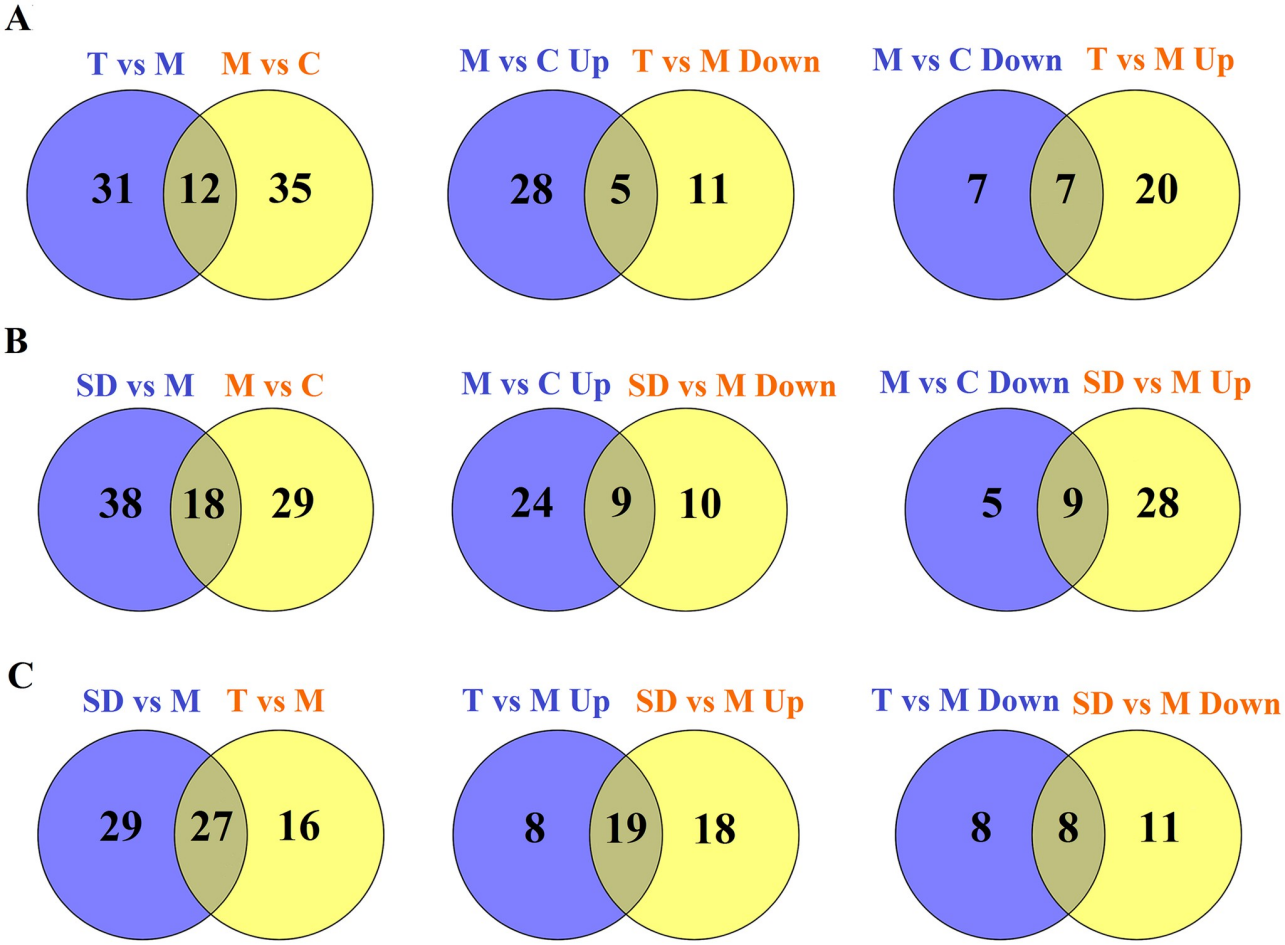
Comparisons of the number and overlapping relationships of differentially expressed proteins between different samples. Yellow circle and purple circle represents number of DEPs in different comparable groups. A: comparable groups: T vs M and M vs C; B: comparable groups: SD vs M and M vs C; C: comparable groups: SD vs M and T vs M.

Further analysis was made to compare the number and overlapping relationships of DE proteins between different groups. As shown in Fig 2A, 12 proteins were commonly changed (opposite direction of change) in M vs C and T vs M, while 31 and 35 DE proteins were exclusive to T vs M and M vs C categories, respectively. Further analysis of shared DE proteins revealed that traditional acupuncture can reverse the up-regulation of 5 proteins and the down-regulation of 7 proteins in the IBS-D model group; see Fig 2A, Table 2. SD vs M and M vs C shared 18 DE proteins, while 38 DE proteins were peculiar to comparing group SD vs M and 29 DE proteins were exclusive to M vs C. Then the 18 shared DE genes were analyzed to identify important proteins related with the effect of syndrome different acupuncture. Fig 2B demonstrated that SD acupuncture can reverse the up-regulation of 9 DE proteins and the down-regulation of 9 DE proteins in the IBS-D model group (Table 2). Likewise, by comparing the DE proteins of T vs M and SD vs M, we identified 27 commonly changed proteins. 29 DE proteins were peculiar to T vs M and 16 were peculiar to SD vs M, see Table 3. The shared 27 DE proteins represent the common effects of traditional acupuncture and syndrome different acupuncture on the protein expression pattern of colonic mucosa in IBS-D rats. Of these, 19 DE proteins were up-regulated and 8 DE proteins were down-regulated in group T and group M compared to control samples; see Fig 2C, Table 2.

**Table 2:** Important shared DE proteins in two comparing groups.

**Uniprot accession**	**Protein name**	**Gene**	**M vs C**		**T vs M**	
			**FC**	**P-value**	**FC**	**P-value**
Q9Z1N4	3(2),5-bisphosphate nucleotidase 1	Bpnt1	1.9	0.036	-2.3	0.017
Q497B0	Omega-amidase NIT2	Nit2	1.6	0.020	-2.0	0.019
P32551	Cytochrome b-c1 complex subunit 2, mitochondrial	Uqcrc2	1.5	0.037	-1.5	0.0086
P15999	ATP synthase subunit alpha, mitochondrial	Atp5a1	1.4	0.029	-1.5	0.0025
P35435	ATP synthase subunit gamma, mitochondrial	Atp5c1	1.3	0.006	-1.4	9.90E-05
P11517	Hemoglobin subunit beta-2	N/A	-4.3	0.038	2.4	0.0069
P35704	Peroxiredoxin-2	Prdx2	-1.8	0.040	1.5	0.0027
P62329	Thymosin beta-4; Hematopoietic system regulatory peptide	Tmsb4x	-1.7	0.044	2.0	0.023
P63036	DnaJ homolog subfamily A member 1	Dnaja1	-1.5	0.034	1.5	0.0029
P0C5H9	Mesencephalic astrocyte-derived neurotrophic factor	Manf	-1.4	0.013	7.1	0.0067
P63324	40S ribosomal protein S12	Rps12	-1.4	0.011	1.6	0.014
Q6P799	Serine—tRNA ligase, cytoplasmic	Sars	-1.2	0.021	1.4	0.037
**Uniprot accession**	**Protein name**	**Gene**	**M vs C**		**SD vs M**	
			**FC**	**P-value**	**FC**	**P-value**
B5DFN2	Adenosylhomocysteinase	Ahcyl1	3.2	0.034	-2.3	0.045
P62744	AP-2 complex subunit sigma	Ap2s1	2.0	0.013	-2.3	0.033
P14173	Aromatic-L-amino-acid decarboxylase	Ddc	1.9	0.022	-1.8	0.017
Q9Z1N4	3(2),5-bisphosphate nucleotidase 1	Bpnt1	1.9	0.036	-3.0	0.0083
P49432	Pyruvate dehydrogenase E1 component subunit beta, mitochondrial	Pdhb	1.7	0.017	-1.4	0.030
P23965	Enoyl-CoA delta isomerase 1, mitochondrial	Eci1	1.5	0.0015	-1.5	0.0010
P32551	Cytochrome b-c1 complex subunit 2, mitochondrial	Uqcrc2	1.5	0.037	-1.6	0.010
P15999	ATP synthase subunit alpha, mitochondrial	Atp5a1	1.4	0.029	-1.5	0.0031
P35435	ATP synthase subunit gamma, mitochondrial	Atp5c1	1.3	0.0068	-1.4	0.00078
P62138	Serine/threonine-protein phosphatase PP1-alpha catalytic subunit	Ppp1ca	-∞	0.00018	+∞	0.027
Q9Z0V5	Peroxiredoxin-4	Prdx4	-1.9	0.024	1.6	0.021
P35704	Peroxiredoxin-2	Prdx2	-1.8	0.040	1.6	0.0086
P62329	Thymosin beta-4; Hema topoietic system regulatory peptide	Tmsb4x	-1.7	0.044	2.3	0.030
P27824	Calnexin	Canx	-1.6	0.043	1.6	0.044
P63036	DnaJ homolog subfamily A member 1	Dnaja1	-1.5	0.034	1.7	0.0090
P0C5H9	Mesencephalic astrocyte-derived neurotrophic factor	Manf	-1.4	0.013	7.1	0.0067
P63324	40S ribosomal protein S12	Rps12	-1.4	0.011	1.6	0.014
Q6P799	Serine—tRNA ligase, cytoplasmic	Sars	-1.2	0.021	1.4	0.037
**Uniprot accession**	**Protein name**	**Gene**	**T vs M**		**SD vs M**	
			**FC**	**P-value**	**FC**	**P-value**
P0C5H9	Mesencephalic astrocyte-derived neurotrophic factor	Manf	7.1	0.0067	8.3	0.0023
Q5M9G3	Caprin-1	Caprin1	3.3	0.033	3.6	0.0073
Q63396	Activated RNA polymer ase II transcriptional coactivator p15	Sub1	3.2	0.012	3.1	0.013
P23785	Granulins; Acrogranin; Granulin-1; Granulin-2; Granulin-3; Granulin-4; Granulin-5; Granulin-6; Granulin-7	Grn	2.5	0.013	2.8	0.0039
P62329	Thymosin beta-4; Hematopoietic system regulatory peptide	Tmsb4x	2.0	0.023	2.3	0.030
Q642C0	DnaJ homolog subfamily C member 8	Dnajc8	2.0	0.047	2.2	0.029
F2Z3T4	Muscleblind-like protein 2	Mbnl2	1.9	0.047	2.2	0.028
Q62636; P62836	Ras-related protein Rap-1b; Ras-related protein Rap-1A	Rap1b; Rap1a	1.8	0.0036	2.1	0.018
P24368	Peptidyl-prolyl cis-trans isomerase B	Ppib	1.7	0.0056	1.8	0.024
Q6B345	Protein S100-A11	S100a11	1.6	0.038	1.9	0.0062
P63324	40S ribosomal protein S12	Rps12	1.6	0.014	1.7	0.0022
P63036	DnaJ homolog subfamily A member 1	Dnaja1	1.5	0.0029	1.7	0.0090
P35704	Peroxiredoxin-2	Prdx2	1.5	0.0027	1.6	0.0086
B0BNA7	Eukaryotic translation initiation factor 3 subunit I	Eif3i	1.5	0.014	1.44	0.015
Q8CFN2; Q9JJL4	Cell division control protein 42 homolog	Cdc42	1.5	0.024	1.4	0.036
Q925G0	RNA-binding protein 3	Rbm3	1.5	0.016	1.8	0.026
Q6RUV5	Ras-related C3 botulinum toxin substrate 1	Rac1	1.5	0.043	1.4	0.028
Q6AXT5	Ras-related protein Rab-21	Rab21	1.4	0.020	1.4	0.037
Q6P799	Serine—tRNA ligase, cytoplasmic	Sars	1.4	0.037	1.2	0.021
Q9Z1N4	3(2),5-bisphosphate nucleotidase 1	Bpnt1	-2.3	0.0173	-3.0	0.0083
P32551	Cytochrome b-c1 complex subunit 2, mitochondrial	Uqcrc2	-1.5	0.0086	-1.6	0.010
P15999	ATP synthase subunit alpha, mitochondrial	Atp5a1	-1.5	0.0025	-1.5	0.0031
P0A9B2	Glyceraldehyde-3-phosphate dehydrogenase A	gapA	-1.5	0.014	-1.5	0.013
Q9ER34	Aconitate hydratase, mitochondrial	Aco2	-1.4	0.029	-1.3	0.028
Q66HF1	NADH-ubiquinone oxidoreductase 75 kDa subunit, mitochondrial	Ndufs1	-1.4	0.0058	-1.4	0.018
P35435	ATP synthase subunit gamma, mitochondrial	Atp5c1	-1.4	9.90E-05	-1.4	0.00079
Q920L2	Succinate dehydrogenase [ubiquinone] flavoprotein subunit, mitochondrial	Sdha	-1.2	0.048	-1.2	0.016

**Table 3:** Protein changes unique to group T and group SD relative to group M.

Uniprot accession	Protein name	Gene	FC	P-value
**T vs M**				
P47728	Calretinin	Calb2	+∞	0.049
P11517	Hemoglobin subunit beta-2	N/A	2.4	0.0070
P24090	Alpha-2-HS-glycoprotein	Ahsg	2.1	0.043
P08932; P01048	T-kininogen 2; T-kininogen 2 heavychain; T-kinin; T-kininogen 2 light chain; T-kininogen 1; T-kininogen 1 heavy chain; T-kinin; T-kininogen 1 light chain	Map1	1.8	0.038
Q924S5	Lon protease homolog, mitochondrial	Lonp1	1.6	0.030
O08629	Transcription intermediary factor 1-beta	Trim28	1.5	0.016
P61959; Q5XIF4	Small ubiquitin-related modifier 2; Small ubiquitin-related modifier 3	Sumo2; Sumo3	1.4	0.022
Q62638; Q92896	Golgi apparatus protein 1	Glg1; GLG1	1.3	0.018
P05696	Protein kinase C alpha type	Prkca	-∞	0.025
P37397	Calponin-3	Cnn3	-∞	0.0021
Q5RK00	39S ribosomal protein L46, mitochondrial	Mrpl46	-∞	0.030
Q497B0	Omega-amidase NIT2	Nit2	-2.0	0.019
P43527	Caspase-1; Caspase-1 subunit p20; Caspase-1 subunit p10	Casp1	-1.8	0.048
Q5XIN6	LETM1 and EF-hand domain-containing protein 1, mitochondrial	Letm1	-1.6	0.00085
Q9JID2; P82471; P50148	Guanine nucleotide-binding protein subunit alpha-11	Gna11	-1.4	0.039
Q62667	Major vault protein	Mvp	-1.3	0.021
**SD vs M**				
P62138; P62136	Serine/threonine-protein phosphatase PP1-alpha catalytic subunit	Ppp1ca; PPP1CA	+∞	0.027
Q4G063	Cysteine-rich with EGF-like domain protein 2	Creld2	6.3	0.028
Q06606; P97594	Granzyme-like protein 2	Mcpt10	3.6	0.031
Q5I0E7	Transmembrane emp24 domain-containing protein 9	Tmed9	2.2	0.048
P13084; P06748	Nucleophosmin	Npm1; NPM1	2.1	0.024
P07632	Superoxide dismutase [Cu-Zn]	Sod1	2.1	0.044
Q4KLL0	Transcription elongation factor A protein 1	Tcea1	1.9	0.040
P38656	Lupus La protein homolog	Ssb	1.9	0.039
O35509; P62494	Ras-related protein Rab-11B; Ras-related protein Rab-11A	Rab11b; Rab11a	1.9	0.029
Q920J4	Thioredoxin-like protein 1	Txnl1	1.6	0.011
P35565; P35564; P27824	Calnexin	Canx	1.6	0.044
Q9Z0V5	Peroxiredoxin-4	Prdx4	1.6	0.021
P38983	40S ribosomal protein SA	Rpsa	1.5	0.040
P09895	60S ribosomal protein L5	Rpl5	1.4	0.032
Q63584; P49755	Transmembrane emp24 domain-containing protein 10	Tmed10; TMED10	1.3	0.037
Q63716	Peroxiredoxin-1	Prdx1	1.3	0.00053
Q6AYK6	Calcyclin-binding protein	Cacybp	1.3	0.027
Q8R491	EH domain-containing protein 3	Ehd3	1.2	0.043
P11661	NADH-ubiquinone oxidoreductase chain 5	Mtnd5	-3.0	0.048
B5DFN2	Adenosylhomocysteinase	Ahcyl1	-2.3	0.045
P62744	AP-2 complex subunit sigma	Ap2s1	-2.3	0.033
P62278	40S ribosomal protein S13	Rps13	-2.0	0.041
P14173	Aromatic-L-amino-acid decarboxylase	Ddc	-1.8	0.017
P46413	Glutathione synthetase	Gss	-1.7	0.043
Q6I7R3	Isochorismatase domain-containing protein 1	Isoc1	-1.6	0.048
P23965	Enoyl-CoA delta isomerase 1, mitochondrial	Eci1	-1.5	0.0010
Q99NA5	Isocitrate dehydrogenase [NAD] subunit alpha, mitochondrial	Idh3a	-1.5	0.00087
P29266	3-hydroxyisobutyrate dehydrogenase, mitochondrial	Hibadh	-1.4	0.048
P49432	Pyruvate dehydrogenase E1 component subunit beta, mitochondrial	Pdhb	-1.4	0.029

### Functional distribution of differentially expressed genes

**Fig 3:**
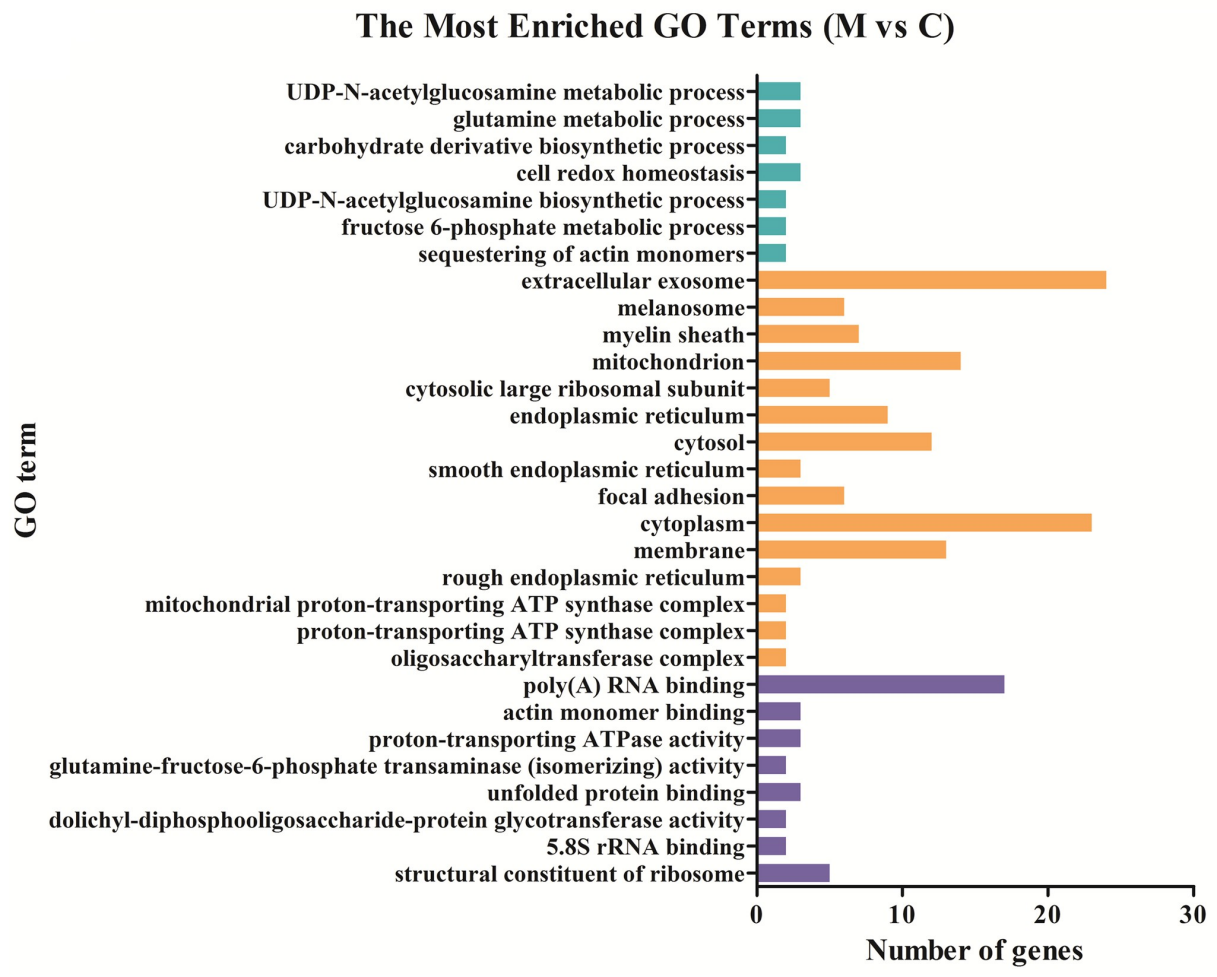
Comparing functional annotations of DEPs between group M and group C. The green bars represent biological process; orange bars represent cellular component; purple bars represent molecular function.

**Fig 4:**
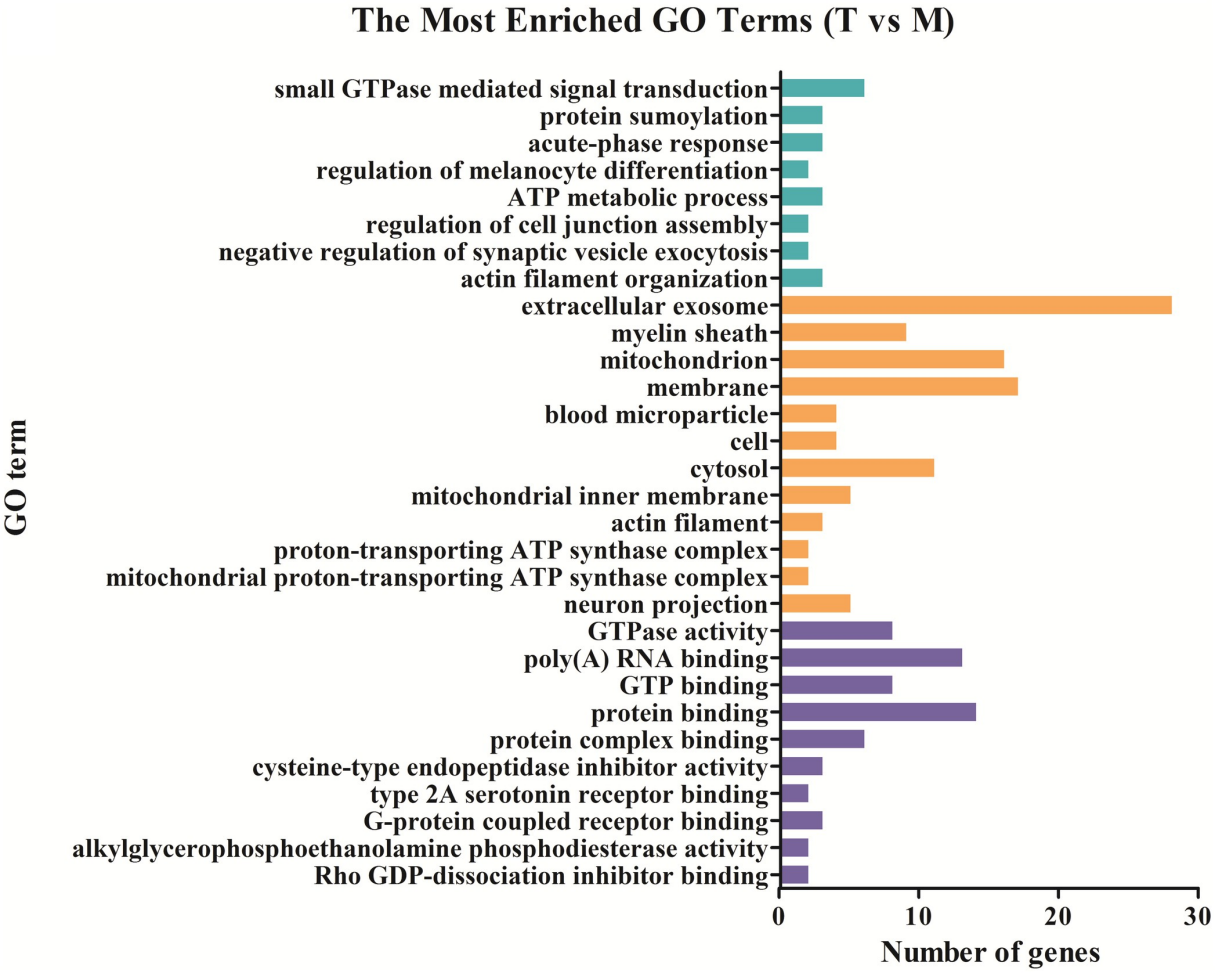
Comparing functional annotations of DEPs between group T and group M. The green bars represent biological process; orange bars represent cellular component; purple bars represent molecular function.

**Fig 5:**
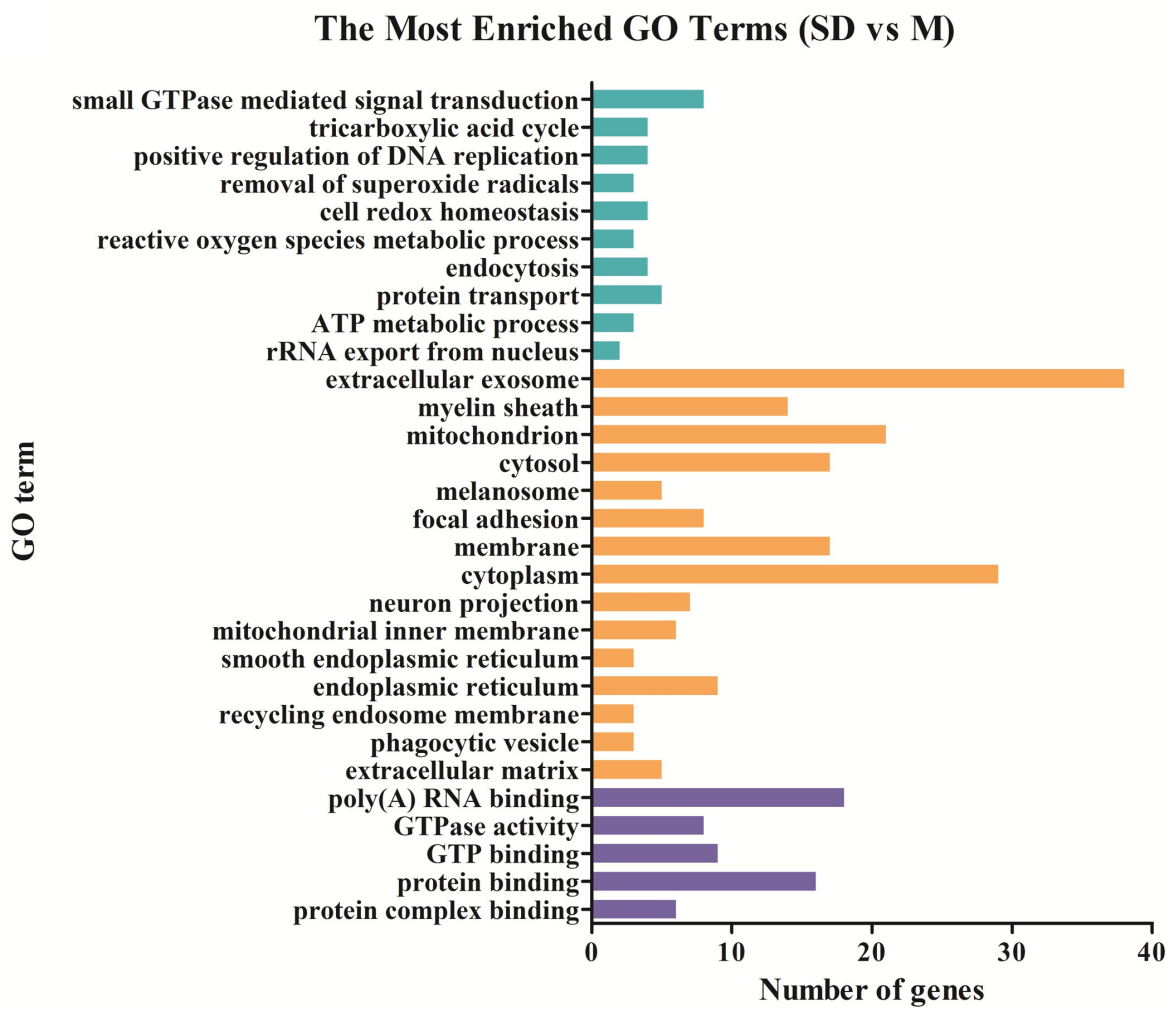
Comparing functional annotations of DEPs between group SD and group M. The green bars represent biological process; orange bars represent cellular component; purple bars represent molecular function.

**Fig 6:**
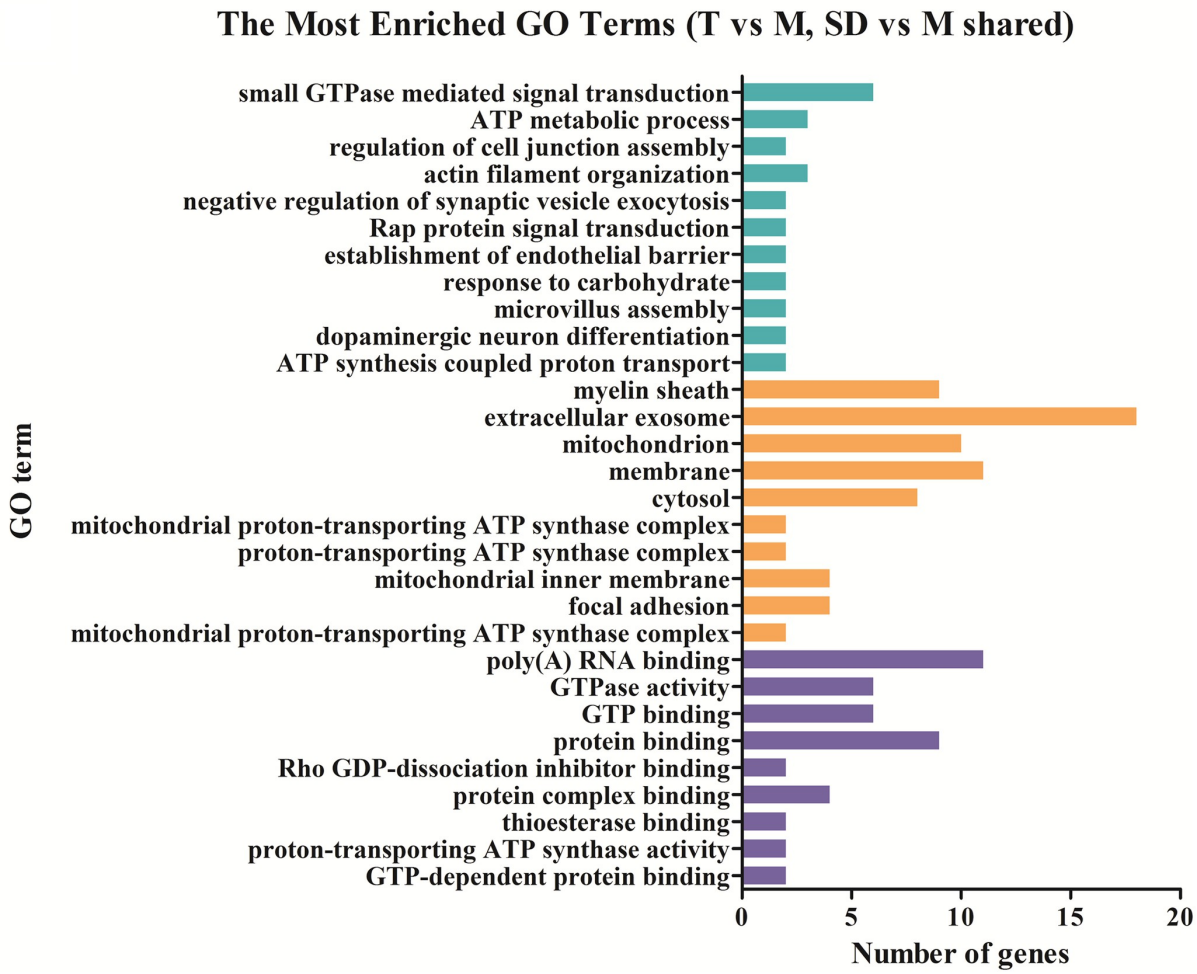
Comparing functional annotations of shared DEPs in comparable groups group T vs group M and group SD vs group M. The green bars represent biological process; orange bars represent cellular component; purple bars represent molecular function.

To detect important proteins related to IBS-D and acupuncture effects, differentially expressed proteins were further analyzed by Gene ontology (GO) term enrichment and Kyoto Encyclopedia of Genes Genomes (KEGG) pathway enrichment to compare functional annotations. The functional distributions of these DE proteins were analyzed in four different sample groups (group 1: M vs C; group 2: T vs M; group 3: SD vs M; group 4: shared DE proteins of T vs M and SD vs M). According to the GO (Gene ontology) categories, the identified DE proteins were categorized into three major functional groups: cellular component, molecular function, and biological process. In group 1, the abundant genes were categorized into 30 major functional groups based on the GO categories; extracellular exosome, cytoplasm, poly (A) RNA binding, mitochondrion and membrane were the top five functional categories (Fig 3). In group 2, DE proteins in the categories of extracellular exosome, membrane, mitochondrion, protein binding and poly (A) RNA binding were the top five functional categories (Fig 4). In group 3, DE proteins in the categories of extracellular exosome, cytoplasm, mitochondrion, poly (A) RNA binding and cytosol were the most abundant functional categories (Fig 5). The 27 shared DE proteins of T vs M and SD vs M were also categorized into 30 major functional groups, among them the top five functional categories were extracellular exosome, poly(A) RNA binding, membrane, mitochondrion and myelin sheath (Fig 6).

**Fig 7:**
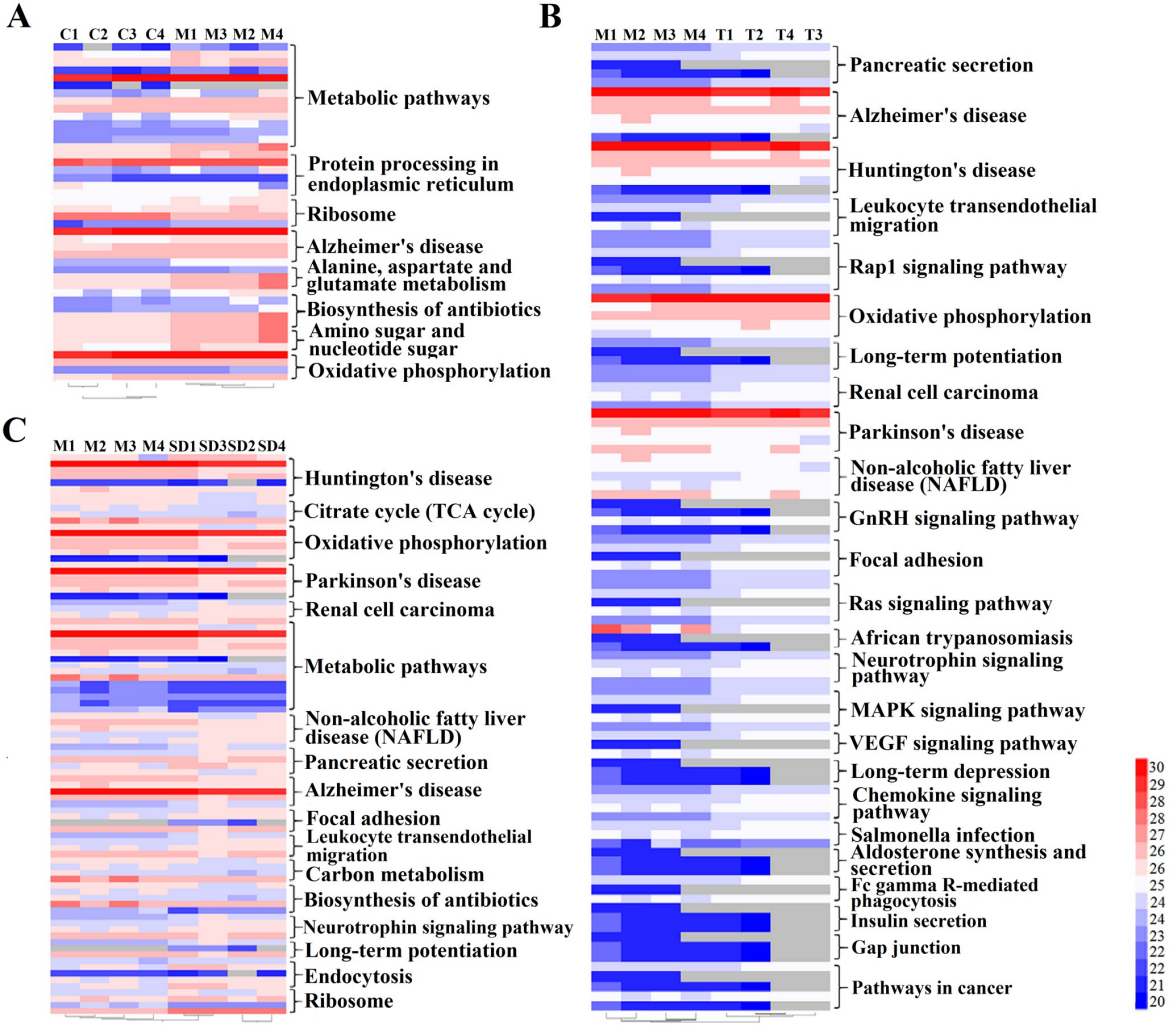
Cluster analysis of differentially expressed proteins and visualization of the changing proteins and their KEGG biological pathways. A: Comparable group: group M and group C; B: Comparable group: group T and group M; C: Comparable group: group SD and group M.

The KEGG pathway enrichments were then performed to categorize the DE proteins in different sample groups. Heatmaps were generated to visualize changing proteins and their biological pathways (Fig 7). In group 1 (M vs C), 10 KEGG pathway terms were identified and the most abundant five terms were: Metabolic pathways, Protein processing in endoplasmic reticulum, Ribosome, Alzheimer’s disease and Alanine, aspartate and glutamate metabolism, as shown in Fig 7A. In group 2 (T vs M), 25 KEGG pathway terms were identified, the most abundant five terms were: Pancreatic secretion, Alzheimer’s disease, Huntington’s disease, Leukocyte transendothelial migration and Rap1 signaling pathway, see Fig 7B. In group 3 (SD vs M), 23 KEGG pathway terms were identified, the most abundant five terms were: Huntington’s disease, Citrate cycle (TCA cycle), Oxidative phosphorylation, Parkinson’s disease and Renal cell carcinoma, see Fig 7C. In group 4, KEGG pathway enrichments were performed to category the 27 DE proteins commonly changed in traditional acupuncture and syndrome different acupuncture compared to model samples. 16 KEGG pathway terms were identified in this group, including Oxidative phosphorylation, Parkinson’s disease, Non-alcoholic fatty liver disease (NAFLD), Renal cell carcinoma, Alzheimer’s disease, Huntington’s disease, Leukocyte transendothelial migration, Neurotrophin signaling pathway, Chemokine signaling pathway, Focal adhesion, Rap1 signaling pathway, Ras signaling pathway, MAPK signaling pathway, Pancreatic secretion and Metabolic pathways.

### Protein-protein interactions and network analysis

**Fig 8:**
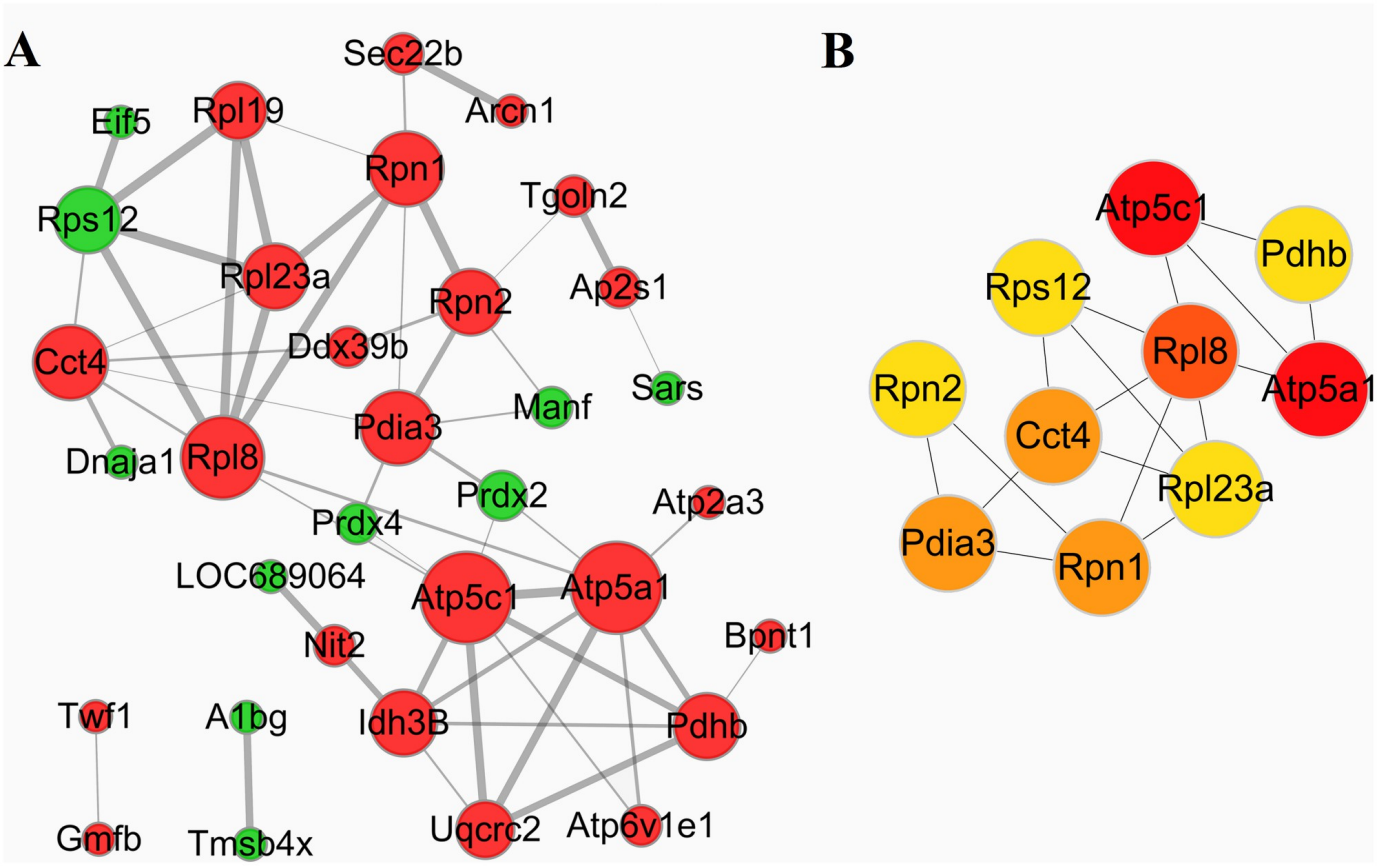
Protein-protein functional network analysis of DEPs between group M and group C. A: The protein-protein functional network. The network was constructed using STRING database and Cytoscape software. B: The top 10 key proteins in the protein-protein interaction networks.

**Fig 9:**
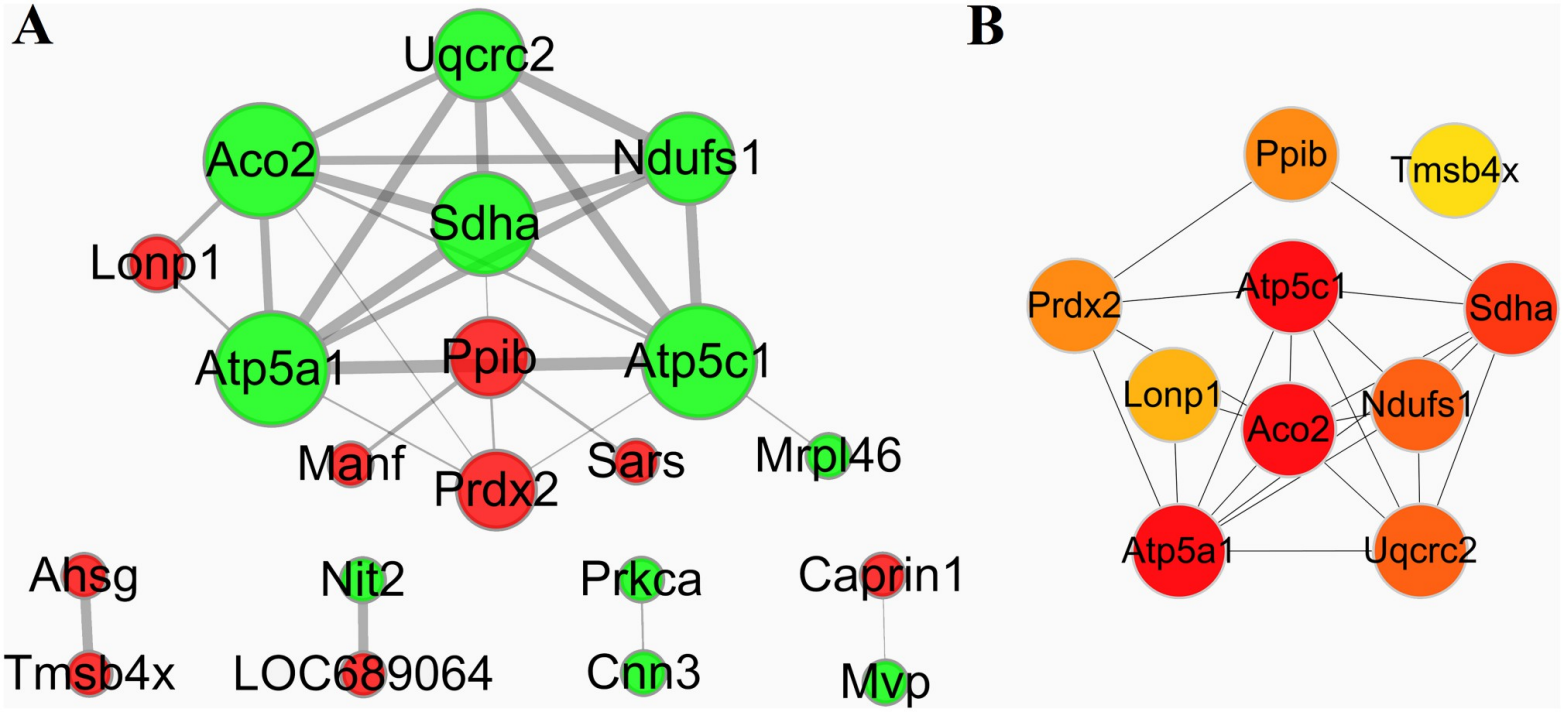
Protein-protein functional network analysis of DEPs between group T and group M. A: The protein-protein functional network. The network was constructed using STRING database and Cytoscape software. B: The top 10 key proteins in the protein-protein interaction networks.

**Fig 10:**
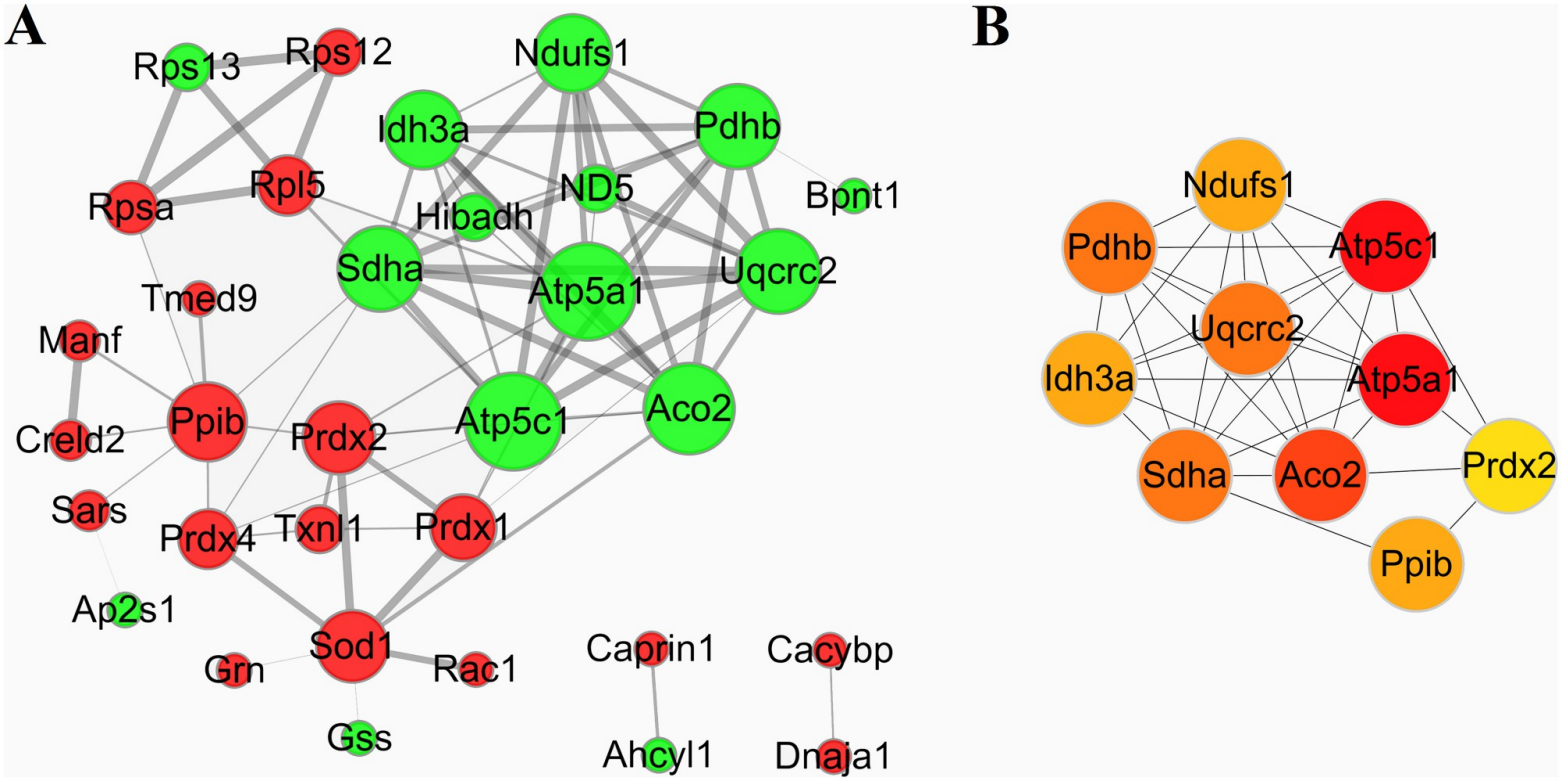
Protein-protein functional network analysis of DEPs between group SD and group M. A: The protein-protein functional network. The network was constructed using STRING database and Cytoscape software. B: The top 10 key proteins in the protein-protein interaction networks.

To investigate the biological interactions among the differentially expressed proteins in response to IBS-D and acupuncture treatment, protein-protein functional network was constructed using STRING database and Cytoscape software. Protein-protein functional networks were analyzed in three sample groups: group 1: M vs C; group 2: T vs M; group 3: SD vs M. As shown in Figs 8A, 9A and 10A, the sizes of edges were determined by the combined_scores of two interacting proteins, the thicker of the edge, the stronger the interaction. The sizes of the nodes were determined by the number of proteins interacting with it; the more of the interacting proteins, the bigger of the node. The color of the nodes represents different regulations of DEPs; the red color means up-regulation and the green color means down-regulation.

In group 1 (M vs C), 33 proteins out of the 47 DEPs were matched and several proteins showed complex interactions with other proteins (Fig 8). The protein functional partnership demonstrated a system-level insight into the effects of IBS-D. MCL clusters identified 11 protein clusters; 2 protein clusters with most gene counts were demonstrated to be highly related to IBS-D. The first group consists of proteins relating energy metabolism, including Atp6v1e1, Idh3B, Atp2a3, Atp5c1, Uqcrc2, Atp5a1, Pdhb and Bpnt1. Expression of DEPs in this clusters were all up-regulated in group M compared with group C, indicating that IBS-D may increase energy expenditure or intensified catabolism. The second group consists of proteins related with the function of ribosomal protein, including Rpl23a, Rpl8, Rpl19, Rps12 and Eif5, as shown in Table 4. Then CytoHubba APP of the Cytoscape software was used to identify key proteins in the Protein-protein interaction network. The top 10 proteins in network M vs C ranked by Degree method were: Atp5c1, Atp5a1, Rpl8, Rpn1, Pdia3, Cct4, Rpn2, Rpl23a, Rps12 and Pdhb, and the sub-network was shown in Fig 8B. The darker color means the more important role the protein plays in the protein-protein interaction network.

**Table 4:** Important MCL clusters of DEPs.

Cluster	Gene	Regulation	Protein Description
M vs C 1	Atp6v1e1	Up	Subunit of the peripheral V1 complex of vacuolar ATPase essential for assembly or catalytic function. V-ATPase is responsible for acidifying a variety of intracellular compartments in eukaryotic cells.
	Idh3B	Up	Sarcoplasmic/endoplasmic reticulum calcium ATPase 3; This magnesium-dependent enzyme catalyzes the hydrolysis of ATP coupled with the transport of the calcium. Transports calcium ions from the cytosol into the sarcoplasmic/endoplasmic reticulum lumen. Contributes to calcium sequestration involved in muscular excitation/contraction.
	Atp2a3	Up	This magnesium-dependent enzyme catalyzes the hydrolysis of ATP coupled with the transport of the calcium. Transports calcium ions from the cytosol into the sarcoplasmic/endoplasmic reticulum lumen. Contributes to calcium sequestration involved in muscular excitation/contraction.
	Atp5c1	Up	ATP synthase subunit gamma, mitochondrial; Mitochondrial membrane ATP synthase produces ATP from ADP in the presence of a proton gradient across the membrane which is generated by electron transport complexes of the respiratory chain.
	Uqcrc2	Up	Cytochrome b-c1 complex subunit 2, mitochondrial; This is a component of the ubiquinol-cytochrome c reductase complex, which is part of the mitochondrial respiratory chain. The core protein 2 is required for the assembly of the complex; Belongs to the peptidase M16 family. UQCRC2/QCR2 subfamily.
	Atp5a1	Up	ATP synthase subunit alpha, mitochondrial; Mitochondrial membrane ATP synthase produces ATP from ADP in the presence of a proton gradient across the membrane which is generated by electron transport complexes of the respiratory chain.
	Pdhb	Up	Pyruvate dehydrogenase E1 component subunit beta, mitochondrial; The pyruvate dehydrogenase complex catalyzes the overall conversion of pyruvate to acetyl-CoA and CO(2), and thereby links the glycolytic pathway to the tricarboxylic cycle.
	Bpnt1	Up	Converts adenosine 3’-phosphate 5’-phosphosulfate (PAPS) to adenosine 5’-phosphosulfate (APS) and 3’(2’)-phosphoadenosine 5’- phosphate (PAP) to AMP. Has 1000-fold lower activity towards inositol 1,4-bisphosphate (Ins(1,4)P2) and inositol 1,3,4-trisphosphate (Ins(1,3,4)P3), but does not hydrolyze Ins1P, Ins(3,4)P2, Ins(1,3,4,5)P4 or InsP6.
M vs C 2	Rpl23a	Up	60S ribosomal protein L23a; This protein binds to a specific region on the 26S rRNA.
	Rpl8	Up	60S ribosomal protein L8; Component of the large ribosomal subunit; Belongs to the universal ribosomal protein uL2 family.
	Rpl19	Up	Ribosomal protein L19.
	Rps12	Down	Ribosomal protein S12-like.
	Eif5	Down	Eukaryotic translation initiation factor 5; Catalyzes the hydrolysis of GTP bound to the 40S ribosomal initiation complex with the subsequent joining of a 60S ribosomal subunit resulting in the release of eIF-2 and the guanine nucleotide. The subsequent joining of a 60S ribosomal subunit results in the formation of a functional 80S initiation complex.
T vs M 1	Aco2	Down	Aconitate hydratase, mitochondrial; Catalyzes the isomerization of citrate to isocitrate via cis-aconitate; Belongs to the aconitase/IPM isomerase family.
	Atp5c1	Down	As shown in “M vs C 1”.
	Uqcrc2	Down	As shown in “M vs C 1”.
	Sdha	Down	Flavoprotein (FP) subunit of succinate dehydrogenase (SDH) that is involved in complex II of the mitochondrial electron transport chain and is responsible for transferring electrons from succinate to ubiquinone (coenzyme Q). Can act as a tumor suppressor.
	Lonp1	Up	Lon protease homolog, mitochondrial; ATP-dependent serine protease that mediates the selective degradation of misfolded, unassembled or oxidatively damaged polypeptides as well as certain short-lived regulatory proteins in the mitochondrial matrix. Participates in the regulation of mitochondrial gene expression and in the maintenance of the integrity of the mitochondrial genome.
	Ndufs1	Down	Core subunit of the mitochondrial membrane respiratory chain NADH dehydrogenase (Complex I) that is believed to belong to the minimal assembly required for catalysis. Complex I functions in the transfer of electrons from NADH to the respiratory chain.
	Atp5a1	Down	As shown in “M vs C 1”.
	Prdx2	Up	Thiol-specific peroxidase that catalyzes the reduction of hydrogen peroxide and organic hydroperoxides to water and alcohols, respectively. Plays a role in cell protection against oxidative stress by detoxifying peroxides and as sensor of hydrogen peroxide-mediated signaling events.
	Mrpl46	Down	Mitochondrial ribosomal protein L46; Belongs to the mitochondrion-specific ribosomal protein mL46 family
SD vs M 1	Aco2	Down	Aconitate hydratase, mitochondrial; Catalyzes the isomerization of citrate to isocitrate via cis-aconitate; Belongs to the aconitase/IPM isomerase family
	Atp5c1	Down	As shown in “M vs C 1”.
	Uqcrc2	Down	As shown in “M vs C 1”.
	Sdha	Down	As shown in “T vs M 1”.
	Hibadh	Down	3-hydroxyisobutyrate dehydrogenase, mitochondrial; 3-hydroxyisobutyrate dehydrogenase.
	Idh3a	Down	Catalytic subunit of the enzyme which catalyzes the decarboxylation of isocitrate (ICT) into alpha-ketoglutarate. The heterodimer composed of the alpha (IDH3A) and beta (IDH3B) subunits and the heterodimer composed of the alpha (IDH3A) and gamma (IDH3G) subunits, have considerable basal activity but the full activity of the heterotetramer requires the assembly and cooperative function of both heterodimers.
	Ndufs1	Down	As shown in “T vs M”.
	Pdhb	Down	As shown in “M vs C 1”.
	Atp5a1	Down	As shown in “M vs C 1”.
	ND5	Down	Core subunit of the mitochondrial membrane respiratory chain NADH dehydrogenase (Complex I) that is believed to belong to the minimal assembly required for catalysis. Complex I functions in the transfer of electrons from NADH to the respiratory chain. The immediate electron acceptor for the enzyme is believed to be ubiquinone.
	Bpnt1	Down	As shown in “M vs C 1”.
SD vs M 2	Gss	Down	Glutathione synthetase; Belongs to the eukaryotic GSH synthase family.
	Grn	Up	Granulins; Granulins have possible cytokine-like activity. They may play a role in inflammation, wound repair, and tissue remodeling.
	Prdx4	Up	Thiol-specific peroxidase that catalyzes the reduction of hydrogen peroxide and organic hydroperoxides to water and alcohols, respectively. Plays a role in cell protection against oxidative stress by detoxifying peroxides and as sensor of hydrogen peroxide-mediated signaling events. Regulates the activation of NF-kappa-B in the cytosol by a modulation of I-kappa-B-alpha phosphorylation.
	Txnl1	Up	Thioredoxin-like protein 1; Active thioredoxin with a redox potential of about -250 mV.
	Prdx1	Up	Peroxiredoxin-1; Thiol-specific peroxidase that catalyzes the reduction of hydrogen peroxide and organic hydroperoxides to water and alcohols, respectively. Plays a role in cell protection against oxidative stress by detoxifying peroxides and as sensor of hydrogen peroxide-mediated signaling events.
	Rac1	Up	Ras-related C3 botulinum toxin substrate 1; Plasma membrane-associated small GTPase which cycles between active GTP-bound and inactive GDP-bound states. In its active state, binds to a variety of effector proteins to regulate cellular responses such as secretory processes, phagocytosis of apoptotic cells, epithelial cell polarization and growth-factor induced formation of membrane ruffles. Rac1 p21/rho GDI heterodimer is the active component of the cytosolic factor sigma 1, which is involved in stimulation of the NADPH oxidase activity in macrophages.
	Prdx2	Up	As shown in “T vs M 1”.
	Sod1	Up	Superoxide dismutase [Cu-Zn]; Destroys radicals which are normally produced within the cells and which are toxic to biological systems; Belongs to the Cu-Zn superoxide dismutase family
SD vs M 3	Rpsa	Up	40S ribosomal protein SA; Required for the assembly and/or stability of the 40S ribosomal subunit. Required for the processing of the 20S rRNA- precursor to mature 18S rRNA in a late step of the maturation of 40S ribosomal subunits. Also functions as a cell surface receptor for laminin. Plays a role in cell adhesion to the basement membrane and in the consequent activation of signaling transduction pathways. May play a role in cell fate determination and tissue morphogenesis. Also acts as a receptor for several other ligands, including the pathogenic prion protein, viruses, and bacteri.
	Rps12	Up	As shown in “M vs C 2”
	Rpl5	Up	60S ribosomal protein L5; Component of the ribosome, a large ribonucleoprotein complex responsible for the synthesis of proteins in the cell. The small ribosomal subunit (SSU) binds messenger RNAs (mRNAs) and translates the encoded message by selecting cognate aminoacyl- transfer RNA (tRNA) molecules. The large subunit (LSU) contains the ribosomal catalytic site termed the peptidyl transferase center (PTC), which catalyzes the formation of peptide bonds, thereby polymerizing the amino acids delivered by tRNAs into a polypeptide chain. The nascent polypeptides leave the ribosome through.
	Rps13	Down	Similar to ribosomal protein S13; Belongs to the universal ribosomal protein uS15 family.

Then we investigate the biological interactions among the DAPs in group 2 (T vs M). 20 proteins out of the 43 DEPs were matched and the protein-protein interaction network was shown in Fig 9A. MCL cluster identified 5 protein clusters; the protein cluster with most gene count consists of proteins related to energy metabolism, including Aco2, Atp5c1, Uqcrc2, Sdha, Lonp1, Ndufs1, Atp5a1, Prdx2 and Mrpl46. Most of the DEPs in this cluster were down-regulated in group T compared with group M (Table 4). Further analysis by CytoHubba revealed the top 10 proteins in network T vs M: Aco2, Atp5a1, Atp5c1, Sdha, Ndufs1, Uqcrc2, Prdx2, Ppib, Lonp1 and Tmsb4x as shown in Fig 9B.

In group 3 (SD vs M), 33 out of 56 DEPs were matched, the protein-protein interaction network was shown in Fig 10A. 8 protein clusters were identified by MCL cluster. 3 protein clusters with most gene counts were demonstrated to be highly related to the effects of syndrome different acupuncture. The first cluster consists of proteins relating energy metabolism, including Aco2, Atp5c1, Uqcrc2, Sdha, Hibadh, Idh3a, Ndufs1, Pdhb, Atp5a1, ND5 and Bpnt1. DEPs in this cluster were all down-regulated in group SD compared with Group M. The second cluster including Gss, Grn, Prdx4, Txnl1, Prdx1, Rac1, Prdx2 and Sod1, most of the DEPs in this cluster were up-regulated in this group, these genes were associated with cell protection against oxidative stress. The third group consists of proteins related with the function of ribosomal protein, including Rpsa, Rps12, Rpl5 and Rps13; most of the DEPs in this cluster were up-regulated in SD samples. The top 10 proteins in network SD vs M analyzed by CytoHubba were: Atp5c1, Atp5a1, Aco2, Uqcrc2, Sdha, Pdhb, Ppib, Idh3a, Ndufs1 and Prdx2, see Fig 10B.

### Identification and verification of the candidate proteins

**Fig 11:**
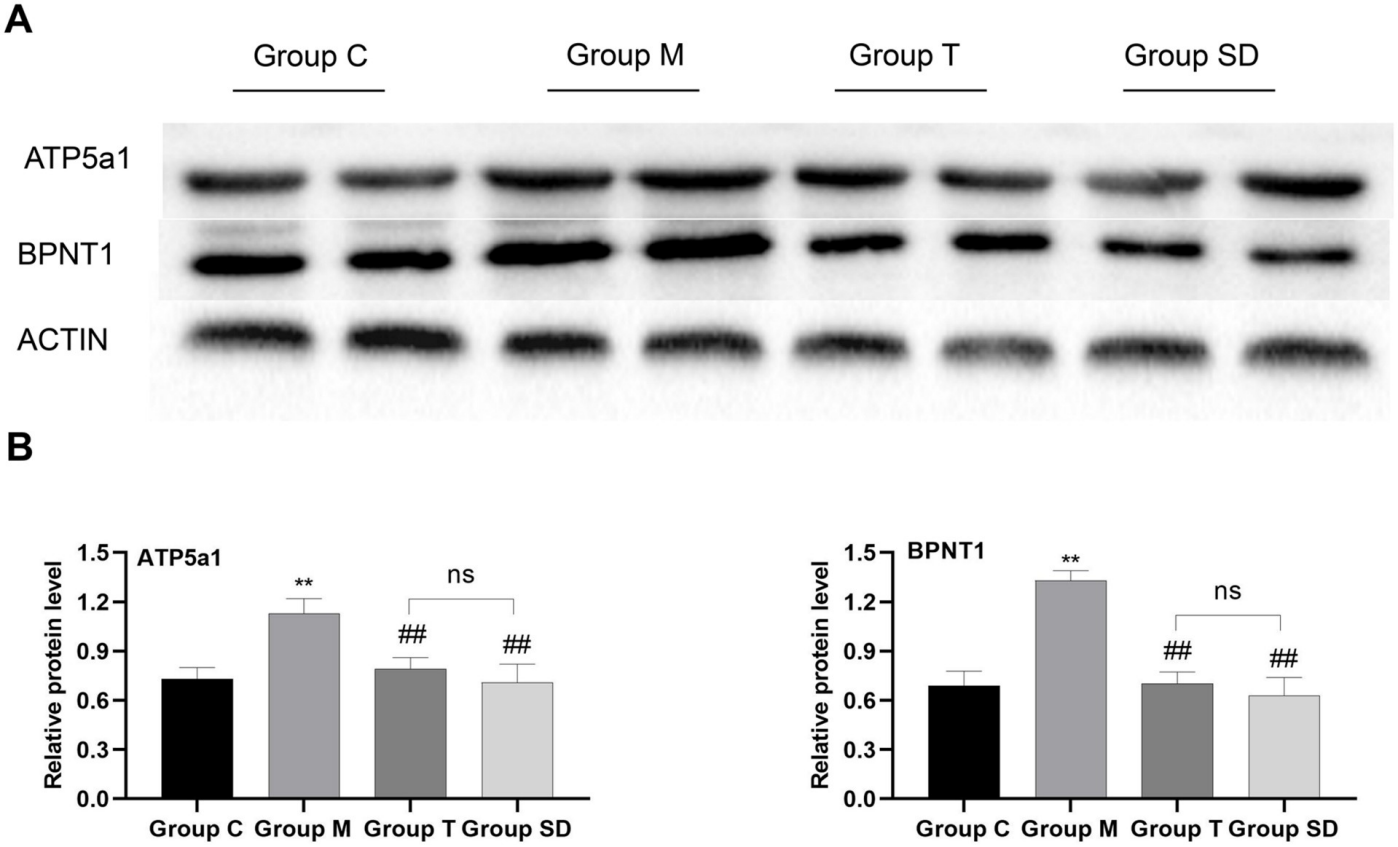
ATP5a1 and BPNT1 expressions in colonic mucosa. The protein level of ATP5a1 and BPNT1 were analyzed by western blot. A. Quantitative analysis of ATP5a1 and BPNT1 expression in colonic mucosa (each group, n = 4, ANOVA). B. All values were expressed as the mean ± SD, **P<0.01: compared with Group C; ##P<0.01: compared with Group M; ns: no significant. Bonferroni’s post hoc test was used for multiple comparisons.

Based on the bioinformatics analysis and the biological function of the differential proteins, two protein candidates were chosen to determine their expression alterations. DEPs Atp5a1 and Bpnt1 were selected as representative proteins and subjected to western blotting, and β-actin (1:2000, Abcam, UK) was used as the internal standard. Atp5a1 and Bpnt1 are key proteins in the protein-protein interaction network and are closely related with IBS-D and acupuncture effects. The results of triplicate western blots for the proteins using protein extracts from the colonic mucosa of rats were consistent with those of the proteomics data (Fig 11).

## Discussion

IBS is the most common functional gastrointestinal disease with a high population prevalence and incidence worldwide. It is characterized by symptoms of chronic abdominal pain and altered bowel habits in the absence of an overtly identifiable cause. IBS generally presents as a complex of symptoms, including psychological dysfunction such as depression and anxiety [22, 29]. There are four IBS subtypes according to the Rome IV criteria: IBS with pain or discomfort and predominant constipation (IBS-C), IBS with diarrhea (IBS-D), mixed IBS, and unsubtyped IBS. IBS-D is the most common subtype among them [1].

The treatment of IBS in modern medicine were mainly psychobehavioral therapy (including psychological suggestion, behavioral therapy, cognitive therapy and hypnotherapy) combined with drug therapy (choose medicine according to symptoms), which aimed to relieve symptoms. However, there is still a lack of specific and effective drugs of IBS and medicine always induce side-effects such as dry eyes and mouth, blurred vision, dizziness and vomiting [8–10]. In view of these, some patients turn to traditional, complementary and integrative medicine. In recent years, acupuncture and related therapies, as well as Chinese herbal medicine, have been extensively used for treating functional gastrointestinal disorders, including IBS, with accurate curative effect [30, 31]. It has been proven that acupuncture treatment can decrease the visceral hypersensitivity, release the visceral pain and improve the abnormal defecation of IBS patients [32–34]. Meta-analysis revealed that the curative effect of acupuncture on IBS is better than medicine, with lower recurrence rate [35, 36]. Therefore, it is of significant scientific value to explore the mechanism of Chinese medicine in prevention and treatment of functional gastrointestinal diseases and illuminate the curative effect of acupuncture in the treatment of IBS.

Traditional Chinese medicine is characterized by the concept of organic wholeness and treatment based on syndrome differentiation. The pathological basis of symptoms, the chemical composition of compounds and the functional route and targets of acupuncture are multiple. Therefore, single factor analytical methods and techniques are not consistent with the concept of organic wholeness in TCM. However, omics technologies can reflect the organ function and metabolism states under the effects of multiple factors from an overall perspective, thus could become a powerful tool in the mechanism research of TCM.

Protein is the final effective factor of cell function and the direct embodiment of life phenomenon. Proteomics, aiming to analyze the proteome and research the carrier of life on a whole level, has become a focus of life science in the era of post-genomics. At present, some research groups have already used proteome technology to explore the mechanism of IBS. Goo et al. performed urinary proteome analysis of the IBS patients subclassified by predominant symptoms using liquid chromatography–tandem mass spectrometry. The results show that the IBS patients subclassified by predominant symptoms showed differences in urine proteome levels. An elevated expression of gelsolin (GSN) in high pain groups and Trefoil Factor 3 (TFF3) levels were higher in IBS groups compared to controls, which can be serve as protein markers as a diagnostic tool in adults with IBS [37]. Anagnostopoulos et al. conducted a proteomic analysis by 2-dimensional gel electrophoresis to identify the protein expression alterations in the serum of IBS patients compared to healthy individuals. Eight significantly different expressed proteins were identified, seven of them were overexpressed in IBS cases and only one was overexpressed in healthy individuals. The results showed a different serum protein profile of IBS patients compared to healthy controls [38]. Li Yijie et al. use a proteomics approach to investigate the molecular mechanism underlying the development of diarrhea-predominant irritable bowel syndrome (IBS-D) with spleen and kidney yang deficiency. The results suggest that SKYD/IBS-D pathophysiology likely involves inflammation, cell growth, apoptosis, stress granule formation, immune activation, loss of epithelial cell integrity, and visceral hypersensitivity [39]. However, the molecular mechanism study of how acupuncture effects on IBS-D based on proteomics have not been totally illustrated yet and need to be further studied.

In the present study, we used a label-free quantitative proteomics technology to survey the global changes in colonic mucosa protein abundance between rats in different groups to elucidate the mechanisms underlying IBS-D disorder and compare the effects of traditional acupuncture and syndrome differentiation acupuncture in treating IBS-D. The IBS-D model rats were obtained using CAS (chronic and acute stress) method. Rats in the SD group received acupuncture stimulation bilaterally at acupoints Neiguan (PC-6), Zusanli (ST 36), Guanyuan (CV 4); and rats in group T received acupuncture stimulation bilaterally at acupoints Zusanli (ST 36), Shangjuxu (ST-37), Tianshu (ST-25) as control. In order to investigate the pathogenic molecular mechanisms of IBS-D and demonstrate the effect of acupuncture treatment, the proteomic analysis based on LC-MS technology was explored. The proteomic data of the colonic mucosa in different groups was compared with each other to demonstrate differences, identify key differentially expressed proteins, which can elucidate the molecular mechanisms of syndrome differentiation acupuncture in treating IBS-D from the proteomic perspective and provide experimental basis for the clinical acupuncture.

Comparison analysis of the proteomics revealed a significant quantitative alteration in protein with differential abundance between different groups. 47 DEPs were identified between group M and group C. To identify important proteins related to the pathogenic mechanisms of IBS-D, DEPs were further analyzed by GO enrichment and KEGG pathway enrichment. IBS-D models showed metabolic alterations and changes in KEGG pathways such as increased energy expenditure and intense catabolism. The protein-protein interaction (PPI) network analysis was performed to investigate the biological interactions among the DEPs in response to IBS-D (Fig 8). MCL clusters identified 11 protein clusters in the PPI network, 2 protein clusters with most gene counts were demonstrated to be highly related to IBS-D. The first group consists of 8 proteins related with energy metabolism; DEPs in this clusters were all up-regulated in group M compared with group C, indicating that IBS-D may increase energy expenditure or intensified catabolism, see Fig 8A and Table 4. Among these energy metabolism related proteins, Idh3B and Atp2a3 are magnesium-dependent enzyme catalyzes the hydrolysis of ATP coupled with the transport of the calcium, transports calcium ions from the cytosol into the sarcoplasmic/endoplasmic reticulum lumen. They all contribute to calcium sequestration involved in muscular excitation/contraction and are interact with each other. Atp5a1 and Atp5c1 are ATP synthase subunit, produces ATP from ADP in the presence of a proton gradient across the membrane which is generated by electron transport complexes of the respiratory chain. Atp5a1 and Atp5c1 supply the catalytic reaction of Idh3B and Atp2a3 with energy. Pdhb links the glycolytic pathway to the tricarboxylic cycle and interact with Atp5a1 and Atp5c1. Many studies of IBS focused on the abnormal of gastrointestinal dynamics. The dysfunctions of colonic motility, the abnormal construction of smooth muscle are important features of IBS and other gastrointestinal disorders. Previous researches suggested that IBS-D patients showed higher contraction frequency of colonic muscle, the acceleration of colon conduction and the functional change of intestinal motion [40–42]. As shown in our study, Idh3B and Atp2a3 are up-regulated in group M compared with group C, which may cause the imbalance of calcium ions (calcium sequestration) and lead to the abnormal muscular excitation/contraction. This may be one of the mechanisms behind the colonic motility dysfunction of IBS-D patients. Further study suggested that both traditional acupuncture and syndrome differentiation acupuncture can reverse the impairments of the normal energy metabolism to some extent, as shown in Table 4 and Figs 9 and 10. Energy metabolism related proteins Idh3a, Atp5a1, Atp5c1, Pdhb, Bpnt1 and Upcrc2 were down-regulated in SD vs M. Idh3a and Idh3b of M vs C 1 cluster are subunits of heterodimer, have considerable basal activity, so Idh3a is also associated with calcium sequestration and muscular excitation. This may be an underlying cause of acupuncture to improve the colonic motility dysfunction.

Then comparisons were made to reveal the effects of acupuncture on the protein expression pattern of colonic mucosa in IBS-D rats. 56 DEPs were identified in SD vs M. Venn analysis demonstrated that SD acupuncture can reverse the up-regulation of 9 DE proteins and the down-regulation of 9 DE proteins in the IBS-D samples (Table 2). Among these proteins, except for proteins involved in energy metabolism and the function of ribosomal protein, Ppp1ca belongs to threonine-protein phosphatase PP1-alpha catalytic subunit, which is essential for cell division, and participates in the regulation of glycogen metabolism, muscle contractility and protein synthesis. This protein was not being detected in the model group, but was expressed in the SD group, which may associate with the regulation of muscle contractility by SD acupuncture. Manf is an endoplasmic reticulum (ER) protein related to the inhibition of cell proliferation and endoplasmic reticulum (ER) stress-induced cell death. Researches revealed that Manf is required for its role in alleviating ER stress and cell toxicity under hypoxic and ER stress conditions, and protects dopamine neurons and cardiomyocytes from ER stress and apoptosis [43, 44]. Tmsb4x plays an important role in the organization of the cytoskeleton. It binds to and sequesters actin monomers (G actin) and therefore inhibits actin polymerization thus associated with the function of intestinal smooth muscle. In comparison group T vs M, 43 DEPs were identified and 12 proteins were commonly changed (opposite direction of change) in M vs C and T vs M (Table 2), including Bpnt1, Uqcrc2, Atp5a1, Atp5c1, Tmsb4x, Manf, Sars which mentioned above. There are 27 commonly changed proteins in T vs M and SD vs M. These DEPs reflect the common effects of syndrome differentiation acupuncture and traditional acupuncture (Table 2).

The PPI network of SD vs M is more complicated than T vs M (Figs 9 and 10). 33 out of 56 DEPs were matched in SD vs M while only 20 of 43 DEPs were matched in T vs M. 8 protein clusters were identified by MCL cluster in SD vs M PPI network, bioinformatics and functional analyses showed a strong link between SD acupuncture effects and energy metabolism and cell protection against oxidative stress. One cluster consists of 11 proteins is related to energy metabolism which described above, DEPs in this cluster were all down-regulated. The changes in this cluster may imply the impairments of the normal energy metabolism in response to IBS-D, and acupuncture can reverse the impairments to some extent. Another cluster including 8 DEPs were up-regulated in SD samples, these genes were associated with inflammation, wound repair, tissue remodeling and cell protection against oxidative stress. Acupuncture analgesia is a traditional Chinese therapy used for treatment of acute and chronic pain which has good clinical efficacy without side-effects [45, 46]. Research shows that the mechanism of acupuncture analgesia is related to the cell protection against oxidative stress [47, 48]. In this study, 8 DEPs associated with cell protection against oxidative stress were up-regulated in SD group compared to model group. Pdrx1, Pdrx2 and Pdrx4 play role in cell protection against oxidative stress by detoxifying peroxides and as sensor of hydrogen peroxide-mediated signaling events. Sod1 destroys radicals which are normally produced within the cells and which are toxic to biological systems and interact with Pdrx1, Pdrx2 and Pdrx4. Grn is granulins which have possible cytokine-like activity and may play a role in inflammation, wound repair, and tissue remodeling. The up-regulating of Pdrx1, Pdrx2, Pdrx4, Sod1 and Grn may be a mechanism of syndrome differential acupuncture in relieving pain, regulating gut dysbiosis and intestinal barrier function when treating IBS patients. The top 10 important proteins in network SD vs M identified by were mainly involved in the two MCL clusters. However, the regulation of protein cluster relating inflammation, wound repair, cell protection against oxidative stress are not shown in T vs M PPI network. In T vs M PPI network, only 5 protein clusters were identified, one significant protein cluster with 9 DEPs was related to energy metabolism. It demonstrated that both traditional acupuncture and syndrome differential acupuncture can reverse the impairments of normal energy metabolism, but the global protein expression of IBS-D samples is more influenced by syndrome differentiation acupuncture.

To sum up, this study first compared and analyzed the differences of protein expression profile in the colonic mucosa of IBS-D rats before and after the acupuncture treatment. Important proteins associated with the pathogenic mechanisms of IBS-D and effects of acupuncture were identified. The results demonstrated that compared with traditional acupuncture, the global protein expression of IBS-D samples is more influenced by syndrome differentiation acupuncture, which contribute to our knowledge on the molecular mechanisms of syndrome differentiation acupuncture towards IBS-D and serve as an experimental basis for clinical applications.

## Supporting information

S1 Fig The process of animal treatment. (PDF)

S1 Raw images (PDF)

## References

[1] Bai T, Xia J, Jiang Y, Cao H, Zhao Y, Zhang L, et al. Comparison of the Rome IV and Rome III criteria for IBS diagnosis: A cross-sectional survey. Journal of gastroenterology and hepatology. 2017;32(5):1018–25. Epub 2016/11/20. doi: 10.1111/jgh.13642 . 27862281

[2] El-Salhy M. Irritable bowel syndrome: diagnosis and pathogenesis. World journal of gastroenterology. 2012;18(37):5151–63. Epub 2012/10/16. doi: 10.3748/wjg.v18.i37.5151 . 23066308 PMC3468846

[3] Holtmann GJ, Ford AC, Talley NJ. Pathophysiology of irritable bowel syndrome. The lancet Gastroenterology & hepatology. 2016;1(2):133–46. Epub 2017/04/14. doi: 10.1016/S2468-1253(16)30023-1 . 28404070

[4] Brzozowski B, Mazur-Bialy A, Pajdo R, Kwiecien S, Bilski J, Zwolinska-Wcislo M, et al. Mechanisms by which Stress Affects the Experimental and Clinical Inflammatory Bowel Disease (IBD): Role of Brain-Gut Axis. Current neuropharmacology. 2016;14(8):892–900. Epub 2016/04/05. doi: 10.2174/1570159x14666160404124127 . 27040468 PMC5333596

[5] Qin HY, Cheng CW, Tang XD, Bian ZX. Impact of psychological stress on irritable bowel syndrome. World journal of gastroenterology. 2014;20(39):14126–31. Epub 2014/10/24. doi: 10.3748/wjg.v20.i39.14126 . 25339801 PMC4202343

[6] Fond G, Loundou A, Hamdani N, Boukouaci W, Dargel A, Oliveira J, et al. Anxiety and depression comorbidities in irritable bowel syndrome (IBS): a systematic review and meta-analysis. European archives of psychiatry and clinical neuroscience. 2014;264(8):651–60. Epub 2014/04/08. doi: 10.1007/s00406-014-0502-z . 24705634

[7] Thakur ER, Holmes HJ, Lockhart NA, Carty JN, Ziadni MS, Doherty HK, et al. Emotional awareness and expression training improves irritable bowel syndrome: A randomized controlled trial. Neurogastroenterology and motility: the official journal of the European Gastrointestinal Motility Society. 2017;29(12). Epub 2017/06/24. doi: 10.1111/nmo.13143 . 28643436 PMC5690851

[8] Tian FJ HX, Chen Y. Research Progress of Traditional Chinese Non Drug Therapy in the Treatment of Irritable Bowel Syndrome. CHINA JOURNAL OF CHINESE MEDICINE. 2013;01:125–27.

[9] Manheimer E, Cheng K, Wieland LS, Min LS, Shen X, Berman BM, et al. Acupuncture for treatment of irritable bowel syndrome. The Cochrane database of systematic reviews. 2012;5(5):Cd005111. Epub 2012/05/18. doi: 10.1002/14651858.CD005111.pub3 . 22592702 PMC3718572

[10] Chao GQ, Zhang S. Effectiveness of acupuncture to treat irritable bowel syndrome: a meta-analysis. World journal of gastroenterology. 2014;20(7):1871–7. Epub 2014/03/04. doi: 10.3748/wjg.v20.i7.1871 . 24587665 PMC3930986

[11] Eisenberg DM, Davis RB, Ettner SL, Appel S, Wilkey S, Van Rompay M, et al. Trends in alternative medicine use in the United States, 1990–1997: results of a follow-up national survey. Jama. 1998;280(18):1569–75. Epub 1998/11/20. doi: 10.1001/jama.280.18.1569 . 9820257

[12] Li H, He T, Xu Q, Li Z, Liu Y, Li F, et al. Acupuncture and regulation of gastrointestinal function. World journal of gastroenterology. 2015;21(27):8304–13. Epub 2015/07/29. doi: 10.3748/wjg.v21.i27.8304 . 26217082 PMC4507100

[13] Ren BB, Yu Z, Xu B. [Overview of the two-way regulatory effect of acupuncture on gastrointestinal motility]. Zhongguo zhen jiu = Chinese acupuncture & moxibustion. 2012;32(8):765–8. Epub 2012/10/18. . 23072106

[14] Iwa M, Nakade Y, Pappas TN, Takahashi T. Electroacupuncture elicits dual effects: stimulation of delayed gastric emptying and inhibition of accelerated colonic transit induced by restraint stress in rats. Digestive diseases and sciences. 2006;51(8):1493–500. Epub 2006/07/27. doi: 10.1007/s10620-006-9083-7 . 16868821

[15] Boltin D, Niv Y. Pharmacological and alimentary alteration of the gastric barrier. Best practice & research Clinical gastroenterology. 2014;28(6):981–94. Epub 2014/12/03. doi: 10.1016/j.bpg.2014.09.001 . 25439065

[16] Shi JJ, Huang LF. [Effects of transcutaneous electrical acupoint stimulation of "Zusanli" (ST 36) on gastric mucosal injury in exercise stress-induced gastric ulcer rats]. Zhen ci yan jiu = Acupuncture research. 2013;38(3):181–5. Epub 2013/09/07. . 24006661

[17] Yang ZB, Yan J, Zou XP, Yi SX, Chang XR, Lin YP, et al. Enhanced expression of epidermal growth factor receptor gene in gastric mucosal cells by the serum derived from rats treated with electroacupuncture at stomach meridian acupoints. World journal of gastroenterology. 2006;12(34):5557–61. Epub 2006/09/29. doi: 10.3748/wjg.v12.i34.5557 . 17007000 PMC4088245

[18] Xing J, Larive B, Mekhail N, Soffer E. Transcutaneous electrical acustimulation can reduce visceral perception in patients with the irritable bowel syndrome: a pilot study. Alternative therapies in health and medicine. 2004;10(1):38–42. Epub 2004/01/20. . 14727498

[19] Bonaz B, Sabate JM. [Brain-gut axis dysfunction]. Gastroenterologie clinique et biologique. 2009;33 Suppl 1:S48–58. Epub 2009/11/07. doi: 10.1016/s0399-8320(09)71525-8 . 19303539

[20] Hui KK, Liu J, Marina O, Napadow V, Haselgrove C, Kwong KK, et al. The integrated response of the human cerebro-cerebellar and limbic systems to acupuncture stimulation at ST 36 as evidenced by fMRI. NeuroImage. 2005;27(3):479–96. Epub 2005/07/28. doi: 10.1016/j.neuroimage.2005.04.037 . 16046146

[21] Zhang YJ YD, Chen XL, Liu ZZ, Tian XM, Wang YF, Yang Q, et al. Principle research of choosing acupoints of IBS acupuncture treatment based on data mining. Lishizhen Medicine and Materia Medica Research. 2020;31:990–3.

[22] Li S, Fei G, Fang X, Yang X, Sun X, Qian J, et al. Changes in Enteric Neurons of Small Intestine in a Rat Model of Irritable Bowel Syndrome with Diarrhea. J Neurogastroenterol Motil. 2016;22(2):310–20. Epub 2015/12/10. doi: 10.5056/jnm15082 . 26645247 PMC4819870

[23] Zheng S XJ, Peng L. Electro-Needling ST36 in IBS Rats: Study on Gastrointestinal Function, CGRP, SP and VIP. Journal of Clinical Acupuncture and Moxibustion. 2018;34:66–9.

[24] Yin CS, Jeong HS, Park HJ, Baik Y, Yoon MH, Choi CB, et al. A proposed transpositional acupoint system in a mouse and rat model. Research in veterinary science. 2008;84(2):159–65. Epub 2007/06/15. doi: 10.1016/j.rvsc.2007.04.004 . 17559895

[25] Minowa T, Ohtsuka S, Sasai H, Kamada M. Proteomic analysis of the small intestine and colon epithelia of adenomatous polyposis coli gene-mutant mice by two-dimensional gel electrophoresis. Electrophoresis. 2000;21(9):1782–6. Epub 2000/06/28. doi: 10.1002/(SICI)1522-2683(20000501)21:9&lt;1782::AID-ELPS1782&gt;3.0.CO;2-3 . 10870965

[26] Perez-Riverol Y, Csordas A, Bai J, Bernal-Llinares M, Hewapathirana S, Kundu DJ, et al. The PRIDE database and related tools and resources in 2019: improving support for quantification data. Nucleic acids research. 2019;47(D1):D442–d50. Epub 2018/11/06. doi: 10.1093/nar/gky1106 . 30395289 PMC6323896

[27] Cox J, Mann M. MaxQuant enables high peptide identification rates, individualized p.p.b.-range mass accuracies and proteome-wide protein quantification. Nature biotechnology. 2008;26(12):1367–72. Epub 2008/11/26. doi: 10.1038/nbt.1511 . 19029910

[28] Deng W, Wang Y, Liu Z, Cheng H, Xue Y. HemI: a toolkit for illustrating heatmaps. PloS one. 2014;9(11):e111988. Epub 2014/11/06. doi: 10.1371/journal.pone.0111988 the authors’ adherence to PLOS ONE Editorial policies and criteria. 25372567 PMC4221433

[29] Li J, Yuan B, Li G, Lu X, Guo Y, Yang Y, et al. Convergent syndromic atrophy of pain and emotional systems in patients with irritable bowel syndrome and depressive symptoms. Neuroscience letters. 2020;723:134865. Epub 2020/02/29. doi: 10.1016/j.neulet.2020.134865 . 32109554

[30] Ho RS WC, Chung VC. Medical synopsis: can acupuncture be an alternative treatment option for patients with refractory functional dyspepsia? Adv Integr Med. 2015;2:143–5.

[31] Chen JD, Yin J, Takahashi T, Hou X. Complementary and Alternative Therapies for Functional Gastrointestinal Diseases. Evidence-based complementary and alternative medicine: eCAM. 2015;2015:138645. Epub 2015/06/13. doi: 10.1155/2015/138645 . 26064152 PMC4434213

[32] Wu IXY, Wong CHL, Ho RST, Cheung WKW, Ford AC, Wu JCY, et al. Acupuncture and related therapies for treating irritable bowel syndrome: overview of systematic reviews and network meta-analysis. Therapeutic advances in gastroenterology. 2019;12:1756284818820438. Epub 2019/02/06. doi: 10.1177/1756284818820438 . 30719074 PMC6348567

[33] Pei L, Chen H, Guo J, Chen L, Wu X, Xu W, et al. Effect of acupuncture and its influence on visceral hypersensitivity in IBS-D patients: Study protocol for a randomized controlled trial. Medicine. 2018;97(21):e10877. Epub 2018/05/26. doi: 10.1097/MD.0000000000010877 . 29794793 PMC6392752

[34] Zhao JM, Lu JH, Yin XJ, Wu LY, Bao CH, Chen XK, et al. Comparison of Electroacupuncture and Mild-Warm Moxibustion on Brain-Gut Function in Patients with Constipation-Predominant Irritable Bowel Syndrome: A Randomized Controlled Trial. Chinese journal of integrative medicine. 2018;24(5):328–35. Epub 2018/05/13. doi: 10.1007/s11655-018-2838-0 . 29752611

[35] MacPherson H, Tilbrook H, Bland JM, Bloor K, Brabyn S, Cox H, et al. Acupuncture for irritable bowel syndrome: primary care based pragmatic randomised controlled trial. BMC gastroenterology. 2012;12:150. Epub 2012/10/26. doi: 10.1186/1471-230X-12-150 . 23095376 PMC3556159

[36] Aleksandar Z BS, Elisa MS, Vahideh T. Treatment of visceral pain associated with irritable bowel syndrome using acupuncture: mechanism of action. World Journal of Traditional Chinese Medicine. 2019;5:181–6.

[37] Goo YA, Cain K, Jarrett M, Smith L, Voss J, Tolentino E, et al. Urinary proteome analysis of irritable bowel syndrome (IBS) symptom subgroups. Journal of proteome research. 2012;11(12):5650–62. Epub 2012/09/25. doi: 10.1021/pr3004437 . 22998556 PMC3631108

[38] Anagnostopoulos Athanassios K, Stamatia Ioakeim, Papadopoulou Aggeliki, et al. Identification of serum proteome signature of irritable bowel syndrome: Potential utility of the tool for early diagnosis and patient’s stratification. Journal of proteomics. 2018;188(30):167–172. doi: 10.1016/j.jprot.2017.07.019 28757466

[39] Li YJ, Su XL, Wu P, Wang JF, Guo Y, Zhu JJ, et al. Proteomics analysis of IBS-D with spleen and kidney yang deficiency. Journal of Traditional Chinese Medical Sciences. 2017;4:39–49.

[40] Spiller R, Aziz Q, Creed F, Emmanuel A, Houghton L, Hungin P, et al. Guidelines on the irritable bowel syndrome: mechanisms and practical management. Gut. 2007;56(12):1770–98. Epub 2007/05/10. doi: 10.1136/gut.2007.119446 . 17488783 PMC2095723

[41] Whitehead WE, Engel BT, Schuster MM. Irritable bowel syndrome: physiological and psychological differences between diarrhea-predominant and constipation-predominant patients. Dig Dis Sci. 1980;25(6):404–13. Epub 1980/06/01. doi: 10.1007/BF01395503 . 7379673

[42] Manabe N, Wong BS, Camilleri M, Burton D, McKinzie S, Zinsmeister AR. Lower functional gastrointestinal disorders: evidence of abnormal colonic transit in a 287 patient cohort. Neurogastroenterol Motil. 2010;22(3):293–e82. Epub 2009/12/23. doi: 10.1111/j.1365-2982.2009.01442.x . 20025692 PMC2852497

[43] Glembotski CC, Thuerauf DJ, Huang CQ, Vekich JA, Gottlieb RA, Doroudgar S. Mesencephalic Astrocyte-derived Neurotrophic Factor Protects the Heart from Ischemic Damage and Is Selectively Secreted upon Sarco/endoplasmic Reticulum Calcium Depletion. J Biol Chem. 2012;287(31):25893–904. doi: 10.1074/jbc.M112.356345 22637475 PMC3406674

[44] Bai MR, Vozdek R, Hnizda A, Jiang CX, Wang BY, Kuchar L, et al. Conserved roles of C. elegans and human MANFs in sulfatide binding and cytoprotection. Nat Commun. 2018;9. ARTN 89710.1038/s41467-018-03355-0. doi: 10.1038/s41467-018-03355-0 29497057 PMC5832864

[45] Sun J LR, Li XY. Observation on curative effect of superficial needling combined with electroacupuncture for trigeminal neuralgia. Shanghai J Acup Moxib. 2020;39:456–61.

[46] Tai ZX FX, Qu S. Intervention effect of electroacupuncture on NF-κB p65 at spinal cord dorsal horn in diabetic neuropathic pain rats. Acta Lab Anim Sci Sin. 2020;28.

[47] Fischer SG, Perez RS, Nouta J, Zuurmond WW, Scheffer PG. Oxidative stress in Complex Regional Pain Syndrome (CRPS): no systemically elevated levels of malondialdehyde, F2-isoprostanes and 8OHdG in a selected sample of patients. International journal of molecular sciences. 2013;14(4):7784–94. Epub 2013/04/12. doi: 10.3390/ijms14047784 . 23574939 PMC3645716

[48] Li XJ YC, Zheng XL. Intervention of electroacupuncture on oxidative stress response from local tissues of a CRPS-I ratmodel. Acta Lab Anim Sci Sin. 2020;28:593–601.

