# Supplementary material for: Proteomic analysis of rat colonic mucosa following acupuncture treatment for irritable bowel syndrome with diarrhea
Source: PLoS One. 2022 Sep 12;17(9):e0273853. doi: 10.1371/journal.pone.0273853 (PMC9467358; doi:10.1371/journal.pone.0273853)
Supplement: S1 Fig — (PDF) [file pone.0273853.s001.pdf]

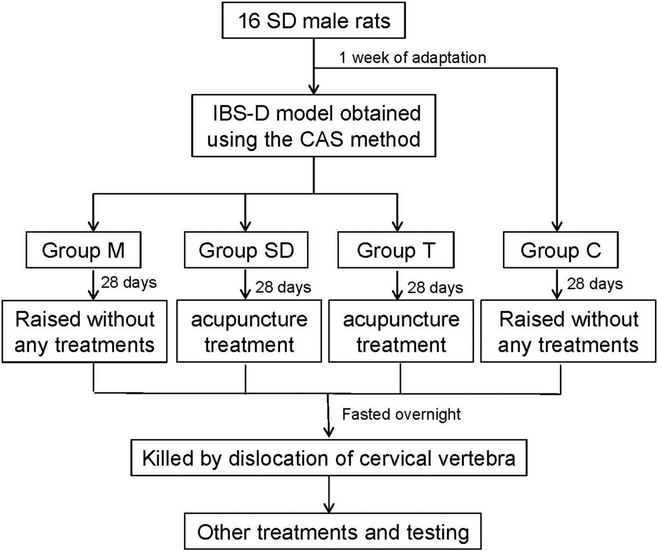
16 SD male rats
1 week of adaptation
IBS-D model obtained using the CAS method
Group M
Group SD
Group T
Group C
28 days
28 days
28 days
28 days
Raised without any treatments
acupuncture treatment
acupuncture treatment
Raised without any treatments
Fasted overnight
Killed by dislocation of cervical vertebra
Other treatments and testing
