# Supplementary figures and images for: Proteomic analysis of rat colonic mucosa following acupuncture treatment for irritable bowel syndrome with diarrhea
Source: PLoS One. 2022 Sep 12;17(9):e0273853. doi: 10.1371/journal.pone.0273853 (PMC9467358; doi:10.1371/journal.pone.0273853)

Actin

Group      C   C   M   M   T   T   SD SD

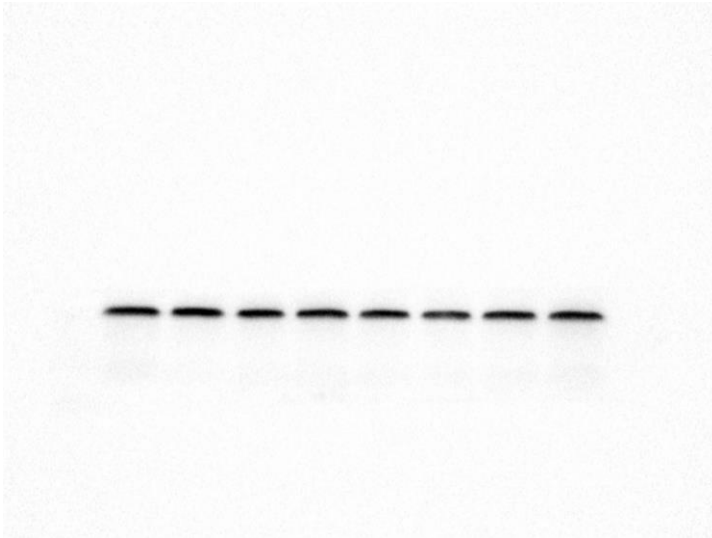

Atp5a1

Group      C   C   C   M   M   T   T   SD   SD

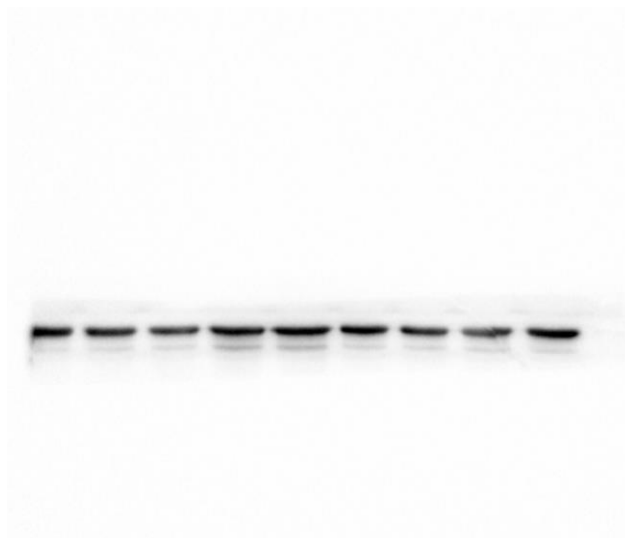

Bpnt1

Group      C   C   M   M   T   T   SD   SD

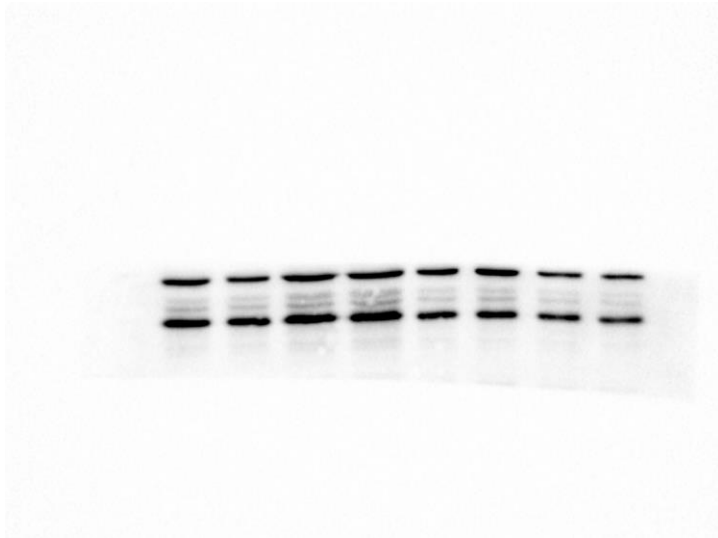

Supplement: S1 Raw images — (PDF) [file pone.0273853.s002.pdf]
